# Longitudinal proteomic investigation of COVID-19 vaccination

**DOI:** 10.1093/procel/pwad004

**Published:** 2023-02-06

**Authors:** Yingrui Wang, Qianru Zhu, Rui Sun, Xiao Yi, Lingling Huang, Yifan Hu, Weigang Ge, Huanhuan Gao, Xinfu Ye, Yu Song, Li Shao, Yantao Li, Jie Li, Tiannan Guo, Junping Shi

**Affiliations:** iMarker Lab, Westlake Laboratory of Life Sciences and Biomedicine, Key Laboratory of Structural Biology of Zhejiang Province, School of Life Sciences, Westlake University, 18 Shilongshan Road, Hangzhou 310024, China; Institute of Basic Medical Sciences, Westlake Institute for Advanced Study, 18 Shilongshan Road, Hangzhou 310024, China; Research Center for Industries of the Future, Westlake University, 600 Dunyu Road, Hangzhou 310030, China; Center for Infectious Disease Research, Westlake University, 18 Shilongshan Road, Hangzhou 310024, China; Department of Translational Medicine Platform, The Affiliated Hospital of Hangzhou Normal University, Hangzhou 310015, China; iMarker Lab, Westlake Laboratory of Life Sciences and Biomedicine, Key Laboratory of Structural Biology of Zhejiang Province, School of Life Sciences, Westlake University, 18 Shilongshan Road, Hangzhou 310024, China; Institute of Basic Medical Sciences, Westlake Institute for Advanced Study, 18 Shilongshan Road, Hangzhou 310024, China; Research Center for Industries of the Future, Westlake University, 600 Dunyu Road, Hangzhou 310030, China; Center for Infectious Disease Research, Westlake University, 18 Shilongshan Road, Hangzhou 310024, China; iMarker Lab, Westlake Laboratory of Life Sciences and Biomedicine, Key Laboratory of Structural Biology of Zhejiang Province, School of Life Sciences, Westlake University, 18 Shilongshan Road, Hangzhou 310024, China; Institute of Basic Medical Sciences, Westlake Institute for Advanced Study, 18 Shilongshan Road, Hangzhou 310024, China; Research Center for Industries of the Future, Westlake University, 600 Dunyu Road, Hangzhou 310030, China; Center for Infectious Disease Research, Westlake University, 18 Shilongshan Road, Hangzhou 310024, China; Westlake Omics (Hangzhou) Biotechnology Co., Ltd., Hangzhou 310024, China; Westlake Omics (Hangzhou) Biotechnology Co., Ltd., Hangzhou 310024, China; Westlake Omics (Hangzhou) Biotechnology Co., Ltd., Hangzhou 310024, China; iMarker Lab, Westlake Laboratory of Life Sciences and Biomedicine, Key Laboratory of Structural Biology of Zhejiang Province, School of Life Sciences, Westlake University, 18 Shilongshan Road, Hangzhou 310024, China; Institute of Basic Medical Sciences, Westlake Institute for Advanced Study, 18 Shilongshan Road, Hangzhou 310024, China; Research Center for Industries of the Future, Westlake University, 600 Dunyu Road, Hangzhou 310030, China; Center for Infectious Disease Research, Westlake University, 18 Shilongshan Road, Hangzhou 310024, China; Westlake Omics (Hangzhou) Biotechnology Co., Ltd., Hangzhou 310024, China; The Fourth School of Clinical Medicine, Zhejiang Chinese Medical University, Hangzhou 310053, China; Department of Translational Medicine Platform, The Affiliated Hospital of Hangzhou Normal University, Hangzhou 310015, China; Medical college of Hangzhou Normal University, Hangzhou 311121, China; Westlake Omics (Hangzhou) Biotechnology Co., Ltd., Hangzhou 310024, China; Department of Infectious Diseases, Nanjing Drum Tower Hospital, The Affiliated Hospital of Nanjing University Medical School, Nanjing 210008, China; Institute of Viruses and Infectious Diseases, Nanjing University, Nanjing 210093, China; iMarker Lab, Westlake Laboratory of Life Sciences and Biomedicine, Key Laboratory of Structural Biology of Zhejiang Province, School of Life Sciences, Westlake University, 18 Shilongshan Road, Hangzhou 310024, China; Institute of Basic Medical Sciences, Westlake Institute for Advanced Study, 18 Shilongshan Road, Hangzhou 310024, China; Research Center for Industries of the Future, Westlake University, 600 Dunyu Road, Hangzhou 310030, China; Center for Infectious Disease Research, Westlake University, 18 Shilongshan Road, Hangzhou 310024, China; Department of Translational Medicine Platform, The Affiliated Hospital of Hangzhou Normal University, Hangzhou 310015, China; Department of Infectious and Hepatology Diseases, The Affiliated Hospital of Hangzhou Normal University, Hangzhou 310015, China

**Keywords:** COVID-19, vaccination, proteomics, neutralizing antibodies (NAbs), machine learning

## Abstract

Although the development of COVID-19 vaccines has been a remarkable success, the heterogeneous individual antibody generation and decline over time are unknown and still hard to predict. In this study, blood samples were collected from 163 participants who next received two doses of an inactivated COVID-19 vaccine (CoronaVac^®^) at a 28-day interval. Using TMT-based proteomics, we identified 1,715 serum and 7,342 peripheral blood mononuclear cells (PBMCs) proteins. We proposed two sets of potential biomarkers (seven from serum, five from PBMCs) at baseline using machine learning, and predicted the individual seropositivity 57 days after vaccination (AUC = 0.87). Based on the four PBMC’s potential biomarkers, we predicted the antibody persistence until 180 days after vaccination (AUC = 0.79). Our data highlighted characteristic hematological host responses, including altered lymphocyte migration regulation, neutrophil degranulation, and humoral immune response. This study proposed potential blood-derived protein biomarkers before vaccination for predicting heterogeneous antibody generation and decline after COVID-19 vaccination, shedding light on immunization mechanisms and individual booster shot planning.

## Introduction

The global public health crisis and the social disruption caused by the coronavirus disease 2019 (COVID-19) pandemic have prompted the emergency use of speedily developed vaccines. As of October 2022, over 12 billion doses had been administered globally (Data source WHO COVID-19 Dashboard accessed on December 16, 2022), although the vaccination distribution is significantly unbalanced (van der Graaf et al., 2022). Previous studies have reported that NAbs responses elicited by an inactivated vaccine (CoronaVac^®^) and an mRNA vaccine (BNT162b2) persisted for 6–8 months after full-schedule vaccination and declined to varying degrees (Falsey et al., 2021; Zeng et al., 2022). Therefore, multiple vaccine boosters and prolonged intervals between vaccine doses are needed to maintain the immunity against SARS-CoV-2 (Zhao et al., 2022), and could induce a robust humoral immune response (Ai et al., 2022). Several studies reported the dynamics of NAbs generation and the molecules dysregulation occurring after vaccinations (Liu et al., 2021; Wang et al., 2022), and potential biomarkers for assessing the effectiveness of vaccination (Ma et al., 2021). Seroconversion rates and antibody titers after COVID-19 vaccines are significantly lower in immunocompromised patients than immunocompetent individuals (Lee et al., 2022), including immune-mediated inflammatory disorders, solid cancers, organ transplant recipients and hematological cancers. While to the best of our knowledge based on literature search, no study has systematically reported heterogeneous hematological host responses to vaccination in both PBMCs and serum. There is currently no known biomarker for predicting the effectiveness of vaccines before vaccination.

In this study, we investigated the host response to Sinovac-CoronaVac^®^. Specifically, we analyzed the proteome of the peripheral blood mononuclear cells (PBMCs) and the sera of a vaccination recipients at different time points. We developed a method to predict the host responses to vaccination. Specifically, we predicted who cannot generate antibodies and whose NAbs tend to disappear earlier than six months after the vaccination. This information would help plan targeted boosters and decide the types and intervals of the vaccinations.

## Results

### Clinical and proteomics profiling before inactivated SARS-CoV-2 vaccination

**Figure 1.**
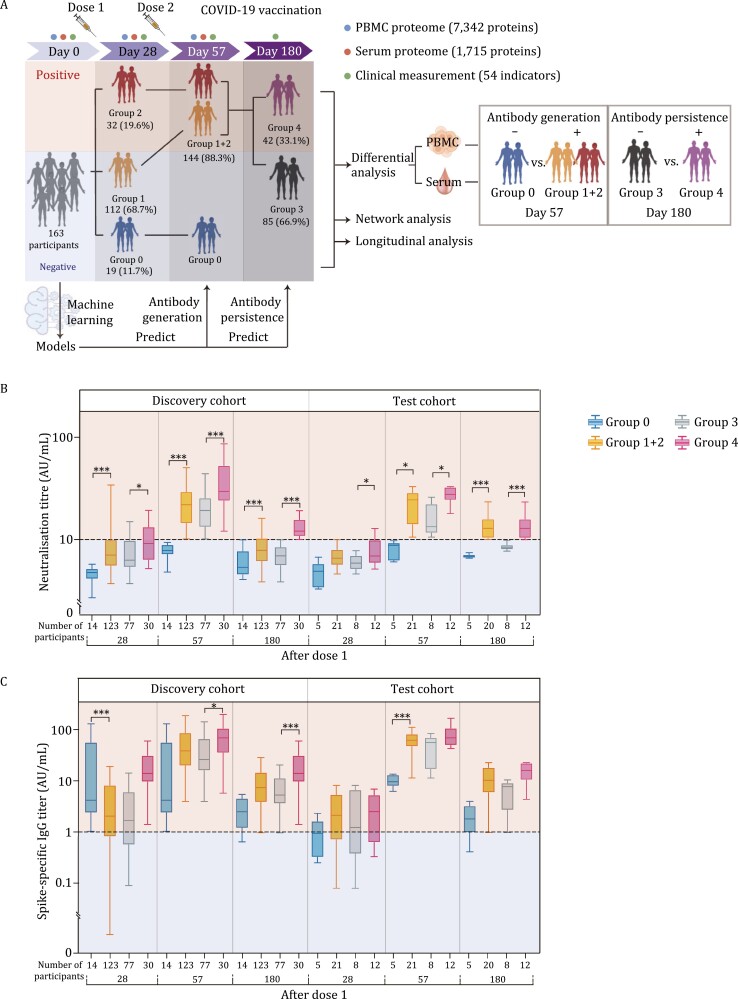
**Study design and overview of clinical indicators.** (A) Study design of the TMT labeling-based quantitative proteomics analysis of the PBMCs and sera samples. Vaccination recipients were vaccinated with two doses of 500 µL CoronaVac^®^, the first at D0 and the second at D28. Blood samples and PBMCs were collected at D0 (before vaccination), D28, and D57. Participants were divided into four groups based on the xenoreactivity of their NAbs on D28 and D57: Group 0, the seronegative group, included the participants that were seronegative on D28 and D57; Group 1, the late seropositive group, included the participants that were seronegative on Day 28 but seropositive on D57; Group 2, the early seropositive group, included the participants that were seropositive on D28 and D57. Groups 1 and 2 were combined into Group 1 + 2 to bring together all the seropositive participants. Group 1 + 2 was then divided into Group 3 (seronegative participants on D180) and Group 4 (the persistently seropositive group, seropositive participants on D180). (B and C) Antibody titers of neutralizing antibodies (B) and Spike-specific IgG (C) to live SARS-CoV-2 at different time points after vaccination. The horizontal line represents the threshold of specific response. The bars represent the median and IQR values of titers. Sample comparisons were tested by Student’s *t*-test or Welch’s *t*-test. ^*^Represents *P* < 0.05, ^**^represents *P* < 0.01, ^***^represents *P* < 0.001.

Between January and February 2021, a total of 163 vaccination recipients were recruited in the discovery (*N* = 137) and the test (*N* = 26) cohorts (Fig. 1A and 1B). The average age was 38.8 years in the discovery cohort and 41.6 years in the test cohort. Besides, most indexes of biochemical and hematology were not significantly different between the two cohorts. More details are shown in Tables 1 and 2, S1, and Fig. S1. All the participants received the first dose of CoronaVac^®^ at day 0 (D0) and the second after 28 days (D28). The qualitative detection of SARS-CoV-2 NAbs and spike-specific IgG was done at D0, D28, day 57 (D57), and day 180 (D180). By D28, 19.6% of all participants (*N* = 32) were NAb seropositive (Group 2, the early seropositive group). By D57, the percentage of seropositive participants reached 88.3% (*N* = 144; Group 1 + 2, which included Group 1, the late seropositive group). The remaining 11.7% (*N* = 19) still had seronegative results (Group 0, the seronegative group). Within Group 1 + 2, 33.1% (*N* = 42) were still positive at D180 (Group 4, the persistently seropositive group), while the remaining ones became seronegative (Group 3) (Fig. 1A). Besides, 10% of participants in Group 0 were IgG seropositive on day 28, which rising to 100% by day 57 and decreasing to 83% by day 180. However, 30% of participants in Group 1 + 2 were IgG seropositive on day 28, which rising to 100% by day 57 and decreasing to 88% by day 180 (Fig. 1C).

**Table 1.** Clinical metadata of the subjects for Group 0 and Group 1 + 2.

Characteristics	Discovery cohort (Cohort 1, *N* = 137)	Test cohort (Cohort 2, *N* = 26)	*P*	Discovery cohort			Test cohort		
				Group 0 (*N* = 14)	Group 1 + 2 (*N* = 123)	*P*	Group 0 (*N* = 5)	Group 1 + 2 (*N* = 21)	*P*
Age (years)	38.839 (11.806)	41.615 (8.782)	0.171	44.643 (12.258)	38.179 (11.622)	0.052	51.800 (5.495)	39.191 (7.633)	0.002
Sex (male/female)	43/94	16/10	0.141	6/8	37/86	0.329	5/0	11/10	0.123
Body mass index (kg/m^2^)	24.026 (3.770)	25.314 (3.138)	0.104	26.002 (4.465)	23.801 (3.636)	0.064	26.208 (4.955)	25.097 (2.676)	0.650
Total bilirubin (μmol/L)	19.883 (6.726)	19.285 (5.663)	0.671	19.650 (5.490)	19.909 (6.871)	0.892	20.080 (6.058)	19.095 (5.706)	0.734
Albumin (g/L)	48.457 (2.302)	47.015 (2.706)	0.005	47.979 (2.555)	48.511 (2.277)	0.414	45.820 (1.587)	47.300 (2.865)	0.281
Alanine aminotransferase (U/L)	20.479 (18.397)	20.704 (10.932)	0.952	17.607 (8.737)	20.806 (19.186)	0.540	19.840 (12.213)	20.910 (10.923)	0.849
Aspartate aminotransferase (U/L)	20.410 (9.150)	22.054 (5.075)	0.375	18.693 (5.496)	20.606 (9.473)	0.461	23.620 (6.460)	21.681 (4.806)	0.454
Alkaline phosphatase (U/L)	66.730 (20.253)	69.462 (17.775)	0.522	67.929 (19.828)	66.594 (20.376)	0.816	72.200 (12.696)	68.810 (18.983)	0.710
γ-Glutamyl transpeptidase (U/L)	24.854 (21.930)	22.692 (12.142)	0.626	19.714 (10.542)	25.439 (22.823)	0.357	21.600 (7.956)	22.952 (13.086)	0.828
LDL-cholesterol (mmol/L)	3.118 (0.779)	3.106 (0.883)	0.941	2.796 (0.705)	3.155 (0.782)	0.102	3.264 (0.108)	3.068 (0.982)	0.665
HDL-cholesterol (mmol/L)	1.372 (0.326)	1.220 (0.268)	0.026	1.266 (0.182)	1.384 (0.337)	0.092	1.284 (0.163)	1.205 (0.288)	0.563
Total cholesterol (mmol/L)	4.965 (0.966)	4.860 (0.983)	0.611	4.557 (0.928)	5.012 (0.963)	0.103	4.984 (0.172)	4.830 (1.094)	0.760
Triglyceride (mmol/L)	1.017 (0.566)	1.174 (0.645)	0.207	1.089 (0.603)	1.009 (0.564)	0.618	0.962 (0.263)	1.225 (0.701)	0.424
Glucose (mmol/L)	4.146 (1.141)	3.502 (0.826)	0.007	4.210 (1.096)	4.138 (1.150)	0.825	3.398 (1.092)	3.526 (0.782)	0.762
Creatinine (μmol/L)	60.307 (29.087)	67.923 (13.702)	0.194	57.786 (17.375)	60.594 (30.169)	0.607	75.800 (13.046)	66.048 (13.47)	0.157
Uric acid (μmol/L)	307.839 (95.341)	343.385 (83.196)	0.078	309.286 (52.889)	307.675 (99.170)	0.952	384.400 (75.075)	333.619 (83.690)	0.227
CRP (mg/L)	1.407 (3.782)	1.206 (1.341)	0.790	1.180 (1.460)	1.442 (3.979)	0.808	1.624 (1.984)	1.235 (1.239)	0.579
Leukocytes (10^9^/L)	6.203 (1.453)	6.692 (1.517)	0.120	2.164 (0.674)	2.063 (0.591)	0.594	6.320 (1.659)	6.781 (1.511)	0.552
Platelets (10^9^/L)	247.796 (57.838)	271.500 (59.567)	0.058	245.143 (62.954)	248.098 (57.496)	0.857	227.200 (53.742)	282.048 (57.010)	0.063
Red blood cells (10^9^/L)	6.203 (1.453)	6.692 (1.517)	0.120	4.742 (0.452)	4.732 (0.630)	0.953	5.320 (0.409)	5.043 (0.605)	0.344
Lymphocytes (10^9^/L)	2.074 (0.598)	2.323 (0.513)	0.048	2.164 (0.674)	2.063 (0.591)	0.552	2.060 (0.541)	2.386 (0.499)	0.209
Hemoglobin (g/L)	140.985 (19.497)	150.039 (20.166)	0.032	138.143 (22.408)	141.309 (19.215)	0.567	160.800 (13.608)	147.476 (20.868)	0.190
Comorbidities, *N* (%)									
Hypertension	13 (9)	6 (23)	0.005	1 (7)	12 (10)	0.092	2 (40)	4 (19)	0.558
T2DM	5 (4)	0 (0)	>0.999	1 (7)	4 (3)	0.426	0 (0)	0 (0)	>0.999
MAFLD	37 (27)	13 (50)	0.020	7 (50)	30 (24)	0.005	2 (40)	11 (52)	>0.999
Seroconversion of neutralizing antibody to live SARS-CoV-2, *N* (%)									
Day 28	30 (22)	2 (7)	0.112	0 (0)	30 (25)	0.040	0 (0)	2 (10)	>0.999
Day 57	123 (90)	21 (81)	0.189	0 (0)	123 (100)	>0.999	0 (0)	21 (100)	<0.001
Day 180	30 (25)^a^	12 (48)^b^	0.021	0 (0) ^c^	30 (28)^d^	>0.999	0 (0) ^e^	12 (60) ^f^	0.043
GMT of neutralizing antibody to live SARS-CoV-2 (AU/mL)									
Day 28	8.523 (5.368)	6.789 (2.781)	0.018	4.817 (1.185)	8.945 (5.497)	<0.001	4.658 (1.379)	7.296 (2.807)	0.055
Day 57	27.372 (28.155)	23.309 (19.138)	0.482	7.746 (1.293)	29.606 (28.884)	<0.001	7.948 (1.64)	26.967 (19.602)	0.043
Day 180	8.627 (4.315)	10.795 (4.325)	0.024	6.055 (1.976)	8.94 (4.422)	<0.001	6.842 (0.343)	11.784 (4.297)	<0.001

Data are shown as mean values (standard deviation within parentheses) and *N* (%). Pearson *χ*^2^ test or Fisher’s exact test were used to analyze the categorical outcomes and Student’s *t*-test or Welch’s *t*-test for continuous outcomes. Hypertension was defined as systolic blood pressure ≥ 140 or diastolic blood pressure ≥ 90 mmHg. T2DM: Type 2 diabetes mellitus, which was defined as fasting glucose ≥ 7.0 mmol/L. MAFLD: metabolic associated fatty liver disease. Superscripts a, b, c, d, e, and f: subjects left in each group were 120, 25, 13, 107, 5, and 20, respectively. Group 0, the seronegative group, included the participants that were seronegative on D28 and D57. Groups 1 + 2 were all the seropositive participants on D57.

**Table 2.** Clinical metadata of the subjects for Group 3 and Group 4.

Characteristics	Discovery cohort (Cohort 3, *N* = 107)	Test cohort (Cohort 4, *N* = 20)	*P*	Discovery cohort			Test cohort		
				Group 3 (*N* = 77)	Group 4 (*N* = 30)	*P*	Group 3 (*N* = 8)	Group 4 (*N* = 12)	*P*
Age (years)	40.019 (10.814)	39.950 (6.970)	0.971	40.130 (10.598)	39.733 (11.531)	0.866	40.625 (6.278)	39.500 (7.634)	0.734
Sex (male/female)	32/75	10/10	0.023	20/57	12/18	0.155	2/6	8/4	0.170
Body mass index (kg/m^2^)	23.965 (3.746)	25.260 (2.638)	0.142	23.879 (3.660)	24.186 (4.014)	0.705	26.738 (1.701)	24.275 (2.745)	0.037
Total bilirubin (μmol/L)	20.114 (7.080)	18.735 (5.604)	0.412	20.705 (6.615)	18.597 (8.075)	0.167	20.913 (6.399)	17.283 (4.736)	0.161
Albumin (g/L)	48.492 (2.245)	47.175 (2.88)	0.023	48.239 (2.191)	49.140 (2.289)	0.062	46.200 (2.183)	47.825 (3.184)	0.226
Alanine aminotransferase (U/L)	21.114 (19.713)	21.310 (11.048)	0.966	18.468 (16.533)	27.907 (25.255)	0.066	19.350 (12.294)	22.617 (10.487)	0.532
Aspartate aminotransferase (U/L)	20.425 (9.418)	21.555 (4.895)	0.602	19.136 (7.126)	23.733 (13.243)	0.08	20.738 (4.525)	22.100 (5.248)	0.556
Alkaline phosphatase (U/L)	66.720 (21.280)	69.000 (19.456)	0.657	66.065 (22.940)	68.400 (16.492)	0.612	59.875 (15.524)	75.083 (19.988)	0.087
γ-Glutamyl transpeptidase (U/L)	26.178 (23.377)	23.3 (13.326)	0.595	24.208 (19.513)	31.233 (31.030)	0.164	18.375 (8.123)	26.583 (15.341)	0.184
LDL-cholesterol (mmol/L)	3.160 (0.800)	3.076 (1.007)	0.680	3.097 (0.769)	3.320 (0.867)	0.196	2.605 (0.655)	3.389 (1.100)	0.088
HDL-cholesterol (mmol/L)	1.370 (0.337)	1.179 (0.27)	0.018	1.406 (0.353)	1.278 (0.276)	0.076	1.188 (0.332)	1.173 (0.235)	0.912
Total cholesterol (mmol/L)	4.996 (0.935)	4.821 (1.122)	0.458	4.907 (0.910)	5.223 (0.973)	0.116	4.316 (0.698)	5.157 (1.247)	0.102
Triglyceride (mmol/L)	1.025 (0.596)	1.245 (0.713)	0.145	0.888 (0.451)	1.375 (0.768)	0.002	1.154 (0.657)	1.305 (0.771)	0.655
Glucose (mmol/L)	4.181 (1.222)	3.593 (0.738)	0.040	4.229 (1.275)	4.060 (1.082)	0.524	3.405 (0.708)	3.718 (0.762)	0.367
Creatinine (μmol/L)	60.009 (31.812)	65.55 (13.621)	0.446	59.610 (37.004)	61.033 (10.440)	0.836	63.000 (10.637)	67.250 (15.51)	0.509
Uric acid (μmol/L)	301.122 (100.483)	331.650 (85.363)	0.205	288.299 (93.366)	334.033 (111.819)	0.034	349.000 (91.377)	320.083 (83.115)	0.473
CRP (mg/L)	1.224 (2.529)	1.136 (1.209)	0.879	1.270 (2.904)	1.106 (1.151)	0.764	1.084 (0.997)	1.170 (1.374)	0.881
Leukocytes (10^9^/L)	6.173 (1.516)	6.815 (1.542)	0.085	6.012 (1.424)	6.587 (1.685)	0.078	6.500 (1.009)	7.025 (1.828)	0.471
Platelets (10^9^/L)	245.738 (60.240)	287.100 (53.450)	0.005	242.351 (60.909)	254.433 (58.591)	0.354	276.500 (53.407)	294.167 (54.621)	0.484
Red blood cells (10^9^/L)	4.719 (0.654)	5.020 (0.611)	0.059	4.631 (0.679)	4.943 (0.533)	0.026	4.675 (0.534)	5.25 0(0.565)	0.035
Lymphocytes (10^9^/L)	2.026 (0.577)	2.395 (0.510)	0.009	1.949 (0.544)	2.223 (0.622)	0.027	2.188 (0.491)	2.533 (0.494)	0.142
Hemoglobin (g/L)	141.037 (20.188)	146.600 (21.01)	0.263	138.558 (21.516)	147.400 (14.776)	0.041	143.375 (14.956)	148.750 (24.647)	0.589
Comorbidity, *N* (%)									
Hypertension	12 (12)	4 (20)	0.280	8 (11)	4 (14)	0.736	0 (0)	4 (34)	0.094
T2DM	4 (4)	0 (0)	>0.999	3 (4)	1 (4)	>0.999	0 (0)	0 (0)	>0.999
MAFLD	30 (28)	11 (55)	0.018	17 (22)	13 (43)	0.028	5 (63)	6 (50)	0.670
Seroconversion of neutralizing antibody to live SARS-CoV-2, *N* (%)									
Day 28	26 (25)	2 (10)	0.240	16 (21)	10 (34)	0.174	0 (0)	2 (17)	0.475
Day 57	107 (100)	20 (100)	>0.999	77 (100)	30 (100)	>0.999	8 (100)	12 (100)	>0.999
Day 180	30 (29)	11 (55)	0.018	0 (0)	30 (100)	<0.001	0 (0)	12 (100)	<0.001
GMT of neutralizing antibody to live SARS-CoV-2 (AU/mL)									
Day 28	9.033 (5.837)	7.41 (2.83)	0.023	8.034 (4.35)	11.596 (8.092)	0.028	6.0563 (1.07254)	8.3125 (3.29792)	0.045
Day 57	29.925 (30.425)	27.133 (20.096)	0.694	24.596 (27.2)	43.603 (34.288)	0.009	15.695 (6.17745)	34.7583 (22.6869)	0.034
Day 180	8.94 (4.422)	11.784 (4.297)	0.009	6.905 (1.641)	14.162 (5.021)	<0.001	8.475 (0.68498)	13.9892 (4.28151)	0.001

Data are shown as mean values (standard deviation in parentheses). Pearson *χ*^2^ test or Fisher’s exact test were used to analyze the categorical outcomes and Student’s *t*-test or Welch’s *t*-test for continuous outcomes. Hypertension was defined as systolic blood pressure ≥ 140 or diastolic blood pressure ≥ 90 mmHg. T2DM: Type 2 diabetes mellitus, which was defined as fasting glucose ≥ 7.0 mmol/L. MAFLD: metabolic associated fatty liver disease. Group 3: the group that became seronegative before D180; Group 4: the group that was seropositive at least until D180.

According to multivariable logistic regression analysis, we found that NAb titers at D28 were positively associated with seropositivity of neutralizing antibodies at D57 after adjusting age, sex, BMI and diastolic blood pressure. Then, we also identified that NAb titers at D28 and D57 could as independent predictors for seropositive of D180 after adjusting covariates (Fig. S1). Blood samples were collected from all participants before their first vaccine dose, then at D28 and D57. Serum and PBMCs were extracted from all blood samples for proteomic profiling.

TMT-based analysis involved 528 samples, including pooled controls for aligning data from different batches to evaluate quantitative accuracy, and technical replicates for evaluating the reproducibility of the assay or technique. These samples were distributed into 33 batches from three time points: D0, D28, and D57. We quantified 7,342 PBMC proteins and 1,715 serum proteins (Table S2; Figs. 1A and S2A). The median coefficients of variance (CV) for the pooled samples were 15.35% and 19.32% for the PBMC and the serum data, respectively (Fig. S2B). The Pearson correlation coefficients of the technical replicates were 98.09% and 96.82% for PBMC and serum, respectively (Fig. S2C). These results showed the robustness of our data and its relatively high consistency and reproducibility.

### Machine learning model for predicting the antibody generation

**Figure 2.**
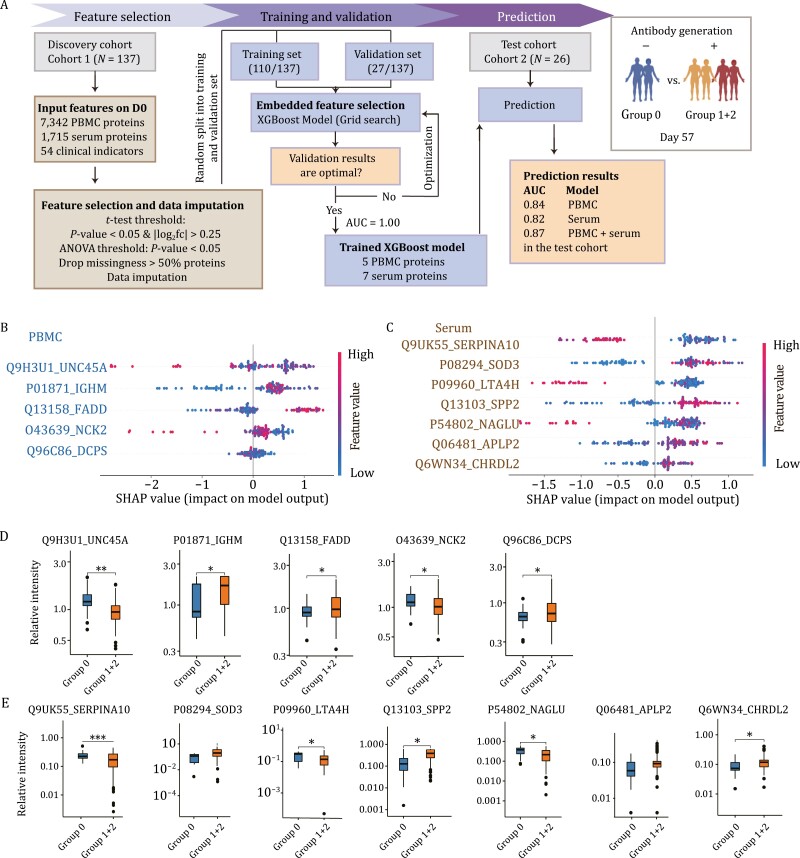
**Machine learning-based prediction of individuals’ seronegative or seropositive status based on their PBMCs and serum proteins before vaccination.** (A) Our machine learning-based predictor was based on PBMC, serum, and both types of proteins. We used the samples from a discovery cohort (Cohort 1, *N* = 137) to optimize the model’s parameters, the discovery dataset was randomly split into a training (80%) and a validation (20%) dataset. The models were then tested using a test cohort (Cohort 2, *N* = 26): the first based on PBMC biomarkers and the second on serum biomarkers. We next developed a third model that was an ensemble of the two previous ones. This third model led to an AUC of 0.87, which was higher than using PBMC or serum proteins individually. (B) The SHAP values of the five PBMC proteins were prioritized using the machine learning model. (C) The SHAP values of the seven serum proteins were prioritized using the machine learning model. (D) Boxplots of the selected biomarker proteins from the PBMC samples. (E) Boxplots of the selected biomarker proteins from the serum samples. Asterisks in (D) and (E) indicate the statistical significance based on the unpaired two-sided Welch’s *t*-test. Specifically, the *P*-values are: ^*^< 0.05; ^**^< 0.01; ^***^< 0.001. Group 0: the seronegative group; Group 1 + 2: the seropositive group.

We next developed a set of models for predicting the seropositivity of individuals 57 days after their first vaccination dose and 28 days after their second one (at D57) based on the proteomics and clinical indicators collected prior to both doses (at D0). Machine learning models were developed using XGBoost (Chen and Guestrin, 2016) (Fig. 2A). Proteins or clinical indicators with a significant difference (*P*-value < 0.05) between the two classes and with |log_2_(fold change)| > 0.25 in the discovery dataset were included in our final feature set. Then, some sparse proteins (NA rate > 50%) were also removed. We optimized the models’ parameters in the discovery dataset (Cohort 1), and generated a model based on the five PBMC proteins and another based on the seven serum proteins. Using the test cohort (Cohort 2), the PBMC model achieved an Areas Under the Curve (AUC) score of 0.84, while the serum one of 0.82 (Fig. 2A). Next, we developed an ensemble model combining these two models, which led to better performance (AUC = 0.87) (Fig. 2A).

Five PBMC proteins (UNC45A, IGHM, FADD, NCK2, and DCPS) and seven serum proteins (SERPINA10, SOD3, LTA4H, SPP2, NAGLU, APLP2, and CHRDL2) were selected for our machine learning models. Most of the above PBMC biomarkers are expressed in immune cells, including B cells, macrophages, natural killer (NK) cells, and dendritic cells (Karlsson et al., 2021). They are thus associated with both innate and adaptive immunity (Fig. 2B and 2D). In particular, unc-45 myosin chaperone A (UNC45A) acts as a co-chaperone for HSP90 promoting progesterone receptor function in the cell, and is required for the NK cell cytotoxicity via lytic granule secretion’s control (Iizuka et al., 2015). Immunoglobulin heavy constant mu (IGHM) is the constant region of immunoglobulin heavy chains and mediates the effector phase of humoral immunity, which eliminates the bound antigens. Fas associated via death domain (FADD) is an adaptor molecule that interacts with various cell surface receptors, mediates cell apoptotic signals, and is essential in early T cell development (Kabra et al., 2001). The seven serum biomarkers are associated with immunity and metabolism (Fig. 2C and 2E): serpin family A member 10 (SERPINA10) and secreted phosphoprotein 2 (SPP2) are secreted proteins associated with coagulation and metabolism; leukotriene A4 hydrolase (LTA4H) is enriched in Kupffer cells, monocytes, and neutrophils; *N*-acetyl-alpha-glucosaminidase (NAGLU) is mainly expressed in most immune cells; superoxide dismutase 3 (SOD3) and chordin like 2 (CHRDL2) can interact with the extracellular matrix (ECM) organization (Karlsson et al., 2021).

Based on our three models, only two participants from the test cohort (Nos. 209 and 233) were mispredicted. Possibly, their predictions were affected by their drug treatments. Specifically, participant No. 209 was incorrectly predicted to be seronegative. This may be due to the long-term treatment with simvastatin and rosuvastatin against hyperlipidemia, which have been suggested to enhance the immune response (Guerra-De-Blas et al., 2019; Karmaus et al., 2019). Participant No. 233, whose atherosclerosis was treated with bisoprolol fumarate before vaccination, was predicted to be seropositive despite being seronegative at D57. Despite these two mispredictions, our results showed that PBMC and serum proteomics could well predict the individual host responses after vaccination.

In addition, predicting those being negative at D28 and then converting (Group 1) or never converting (Group 0) could allow for the earlier switch to another vaccine. Similarly, we developed a set of models based on proteins at D0 and achieved an AUC score of 0.842 (PBMC model), 0.847 (serum model) and 0.853 (ensemble model combining these two models) to predict the individual host responses after vaccination (Fig. S3A–C).

### Increased innate and adaptive immunity in the seropositive group

**Figure 3.**
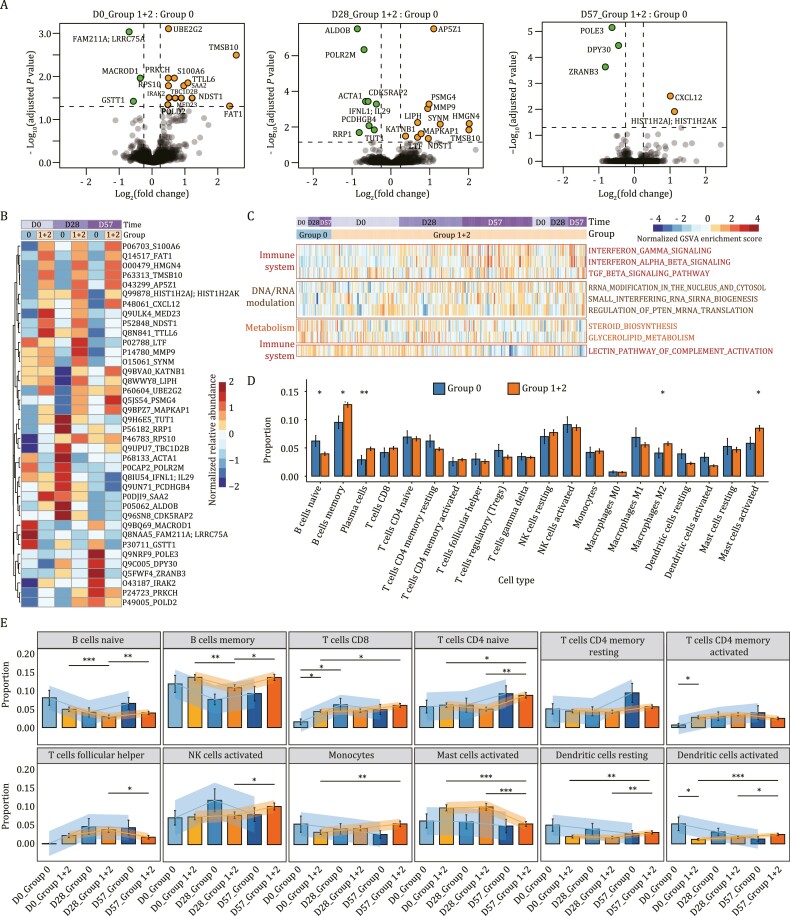
**Comparison of the immune responses in the seropositive and the seronegative groups using the PBMCs proteome.** (A) Identification of NAb status-associated proteins in PBMC using volcano plot analysis on D0, D28, and D57 (two-sided unpaired Welch’s *t*-test). Proteins with B-H adjusted *P*-value < 0.05 with |log_2_(fold change)| > 0.25 were considered as significantly differential expression. (B) Heatmap of the proteins that were significantly regulated in (A). The expression of each protein is shown for both immune response groups and at D0, D28, and D57. (C) Heatmap of the most significantly differentially enriched pathways between the seropositive and the seronegative groups generated using GSVA [B-H adjusted *P*-value < 0.05, |log_2_(fold change)| > 0.25]. (D) Barplots visualizing the inferred proportions (mean ± standard error of mean) of 20 immune cell types. (E) Barplots visualizing the average proportions (mean ± standard error of mean) of B cells, T cells, and several innate immune cells, in the seronegative (Group 0) and seropositive (Group 1 + 2) groups at three time points. Asterisks in (D) and (E) indicate the statistical significance based on the Mann–Whitney rank test. *P*-value: ^*^< 0.05; ^**^< 0.01; ^***^< 0.001.

We next explored the differences between the seropositive and the seronegative groups using the PBMC data. Thirty-eight proteins were differentially expressed (DEPs) within the PBMC proteome between the two groups at three time points [Benjamini-Hochberg (B-H) adjusted *P*-value < 0.05, |log_2_(fold change)| > 0.25] (Table S3; Fig. 3A and 3B). In particular, 33 proteins were dysregulated at D0 or D28. This result suggests that the immune system of the two groups was different at baseline (D0) and was most strongly activated during the early stage after vaccination.

A gene-set variation analysis (GSVA) was then used to identify the most significantly enriched pathways in the seropositive and the seronegative groups. The resulting pathways [B-H adjusted *P*-value < 0.05, |log_2_(fold change)| > 0.25] were mainly involved in the immune system. They included the IFNγ, IFNα and IFNβ signaling, RNA and DNA modulation, and metabolic pathways. Most of these pathways were upregulated in the seropositive group (Fig. 3C).

The DEPs among the three immune response groups—Group 0, Group 1, and Group 2 (Fig. 1A)—were primarily involved in RNA metabolism, cellular processes, and cytoskeleton regulation-related pathways (Fig. S4A–D). Therefore, the dysregulation of these proteins may have contributed to elevated immunity. This agrees with the functional analysis between the seropositive and the seronegative groups. Over 80% of DEPs observed between Group 1 and Group 0 were also detected in the comparison between seropositive (Group 1 + 2) and seronegative (Group 0) groups (Fig. S3D and S3E; Table S3).

We next analyzed the immune cells composition of the PBMCs in our experiment using the deconvolution algorithm of CIBERSORT (Newman et al., 2015). The seropositive group showed an increase in memory B cells and a reduction of naïve B cells (Fig. 3D). In addition, CD8^+^ T cells and activated memory CD4^+^ T cells were significantly higher in the seropositive group than in the seronegative one at D0. The adaptive immune responses were thus significantly enhanced at the baseline in the seropositive group (Fig. 3E). At D57, the memory B cells and the CD8^+^ T cells showed an upward trend over time and were significantly higher in the seropositive group. This result is consistent with the reported host responses to SARS-CoV-2 infection and vaccination (Chen et al., 2022; Sette and Crotty, 2021). Furthermore, some innate immune cells, such as monocytes, activated NK cells, and activated dendritic cells, also increased over time in the seropositive group (Fig. 3E). Moreover, the early seropositive group showed increased memory B cells, activated NK cells, M1 and M2 macrophages (Fig. S4F–G). These results show that the proportion of SARS-CoV-2-specific memory lymphocytes may increase after vaccination with CoronaVac^®^.

### The interaction between metabolism and immunity is linked with seroconversion

**Figure 4.**
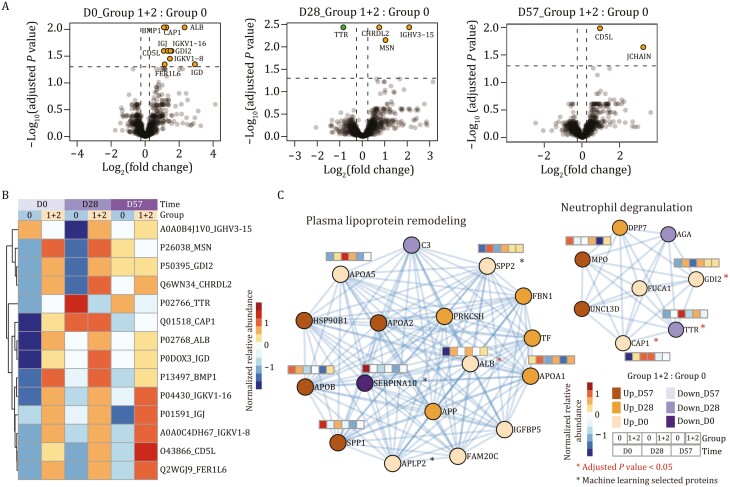
**Comparison of the immune responses in the seropositive and the seronegative groups using the serum proteome.** (A) Identification of NAb status-associated proteins in serum using volcano plot analysis on D0, D28, and D57 by the two-sided unpaired Welch’s *t*-test. Proteins with B-H adjusted *P*-value < 0.05 with |log_2_(fold change)| > 0.25 were considered as significantly differential expression. (B) Heatmap of the proteins that were significantly regulated in (A). The expression of each protein is shown for both immune response groups and at D0, D28, and D57. (C) The most significantly enriched networks generated using significantly dysregulated proteins from the serum proteome. Proteins involved in plasma lipoprotein remodeling and neutrophil degranulation are shown with their expression levels in the seropositive and the seronegative groups at three time points. The cut-off of the dysregulated proteins was set at *P*-value < 0.05 and |log_2_(fold change)| > 0.25. The proteins highlighted with a red * had B-H adjusted *P*-values < 0.05, while those with a black * were selected from our optimized machine learning models. Group 0: the seronegative group; Group 1 + 2: the seropositive group.

We next investigated the differences between the seropositive and the seronegative groups using the serum data. A total of 13 DEPs were found at D0 and D28, and two at D57 [B-H adjusted *P*-value < 0.05, |log_2_(fold change)| > 0.25] (Table S3; Fig. 4A and 4B). In line with our findings from the PBMC data, more DEPs were identified at D0 and D28 than at D57. All the DEPs were upregulated in the seropositive group except transthyretin (TTR). Misfolding and aggregation of TTR, causing amyloid thyroxine protein amyloidosis, has been reported associated with a higher risk of COVID-19 morbidity and mortality (Brannagan et al., 2021). This finding is consistent with our results, as TTR was downregulated in the seropositive group. Many of our serum DEPs are secreted proteins and include components of the immunoglobulin family: IGKV1-8, IGKV1-16, and IGHV3-15 (Schroeder and Cavacini, 2010).

Further functional analyses were performed on the DEPs between the seropositive and the seronegative groups, and among the three immune response groups. The significantly enriched functions were neutrophil degranulation, acute phase response signaling, and hemostasis (Figs. 4B, 4C, S5A and S5B; Table S4). It has been shown that the enriched apolipoprotein family could induce the activation of leukocytes, especially the degranulation of neutrophils (Botham and Wheeler-Jones, 2013). Our analyses showed that 15 out of the 17 proteins involved in plasma lipoprotein remodeling and six out of the eight proteins involved in neutrophil degranulation were upregulated in the seropositive groups (Fig. 4C). This finding suggests that the interaction of metabolism and immunity is closely linked with seroconversion.

### Predicting individual antibody persistence to guide booster shot planning

**Figure 5.**
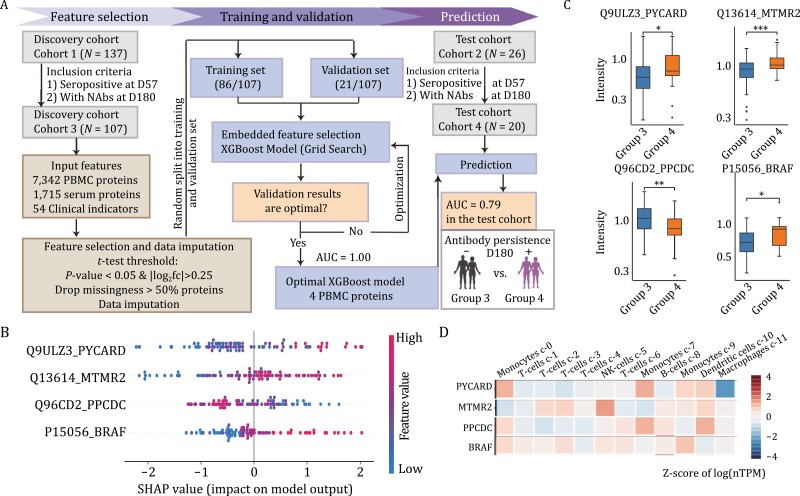
**Proteomics of seropositive and seronegative individuals 180 days after CoronaVac** ^®^ **vaccination.** (A) Workflow for generating a model to predict the antibody persistence till D180. We discarded participants from Group 0 (the seronegative ones) and those without clinical indicators on D180, the remaining two cohorts: a training cohort (Cohort 3, *N* = 107) and a test cohort for the validation (Cohort 4, *N* = 20). (B) SHAP values of the machine learning classifier trained with selected PBMC proteins. (C) Expression of the selected proteins from the PBMC samples. The asterisks indicate the statistical significance based on the unpaired two-sided Welch’s *t*-test. *P*-value: ^*^< 0.05; ^**^< 0.01; ^***^< 0.001. Group 3: the seronegative group on D180; Group 4: the persistently seropositive group. (D) Relative expression of the proteins selected for our model in the different cell type clusters of PBMCs [data from the Human Proteins Atlas (Karlsson et al., 2021)].

To predict whether the antibodies produced after the CoronaVac^®^ vaccination could last for at least 180 days, we generated machine learning models based on the proteomics data and the clinical indicators collected prior to vaccination. In this analysis, we excluded participants from Group 0 (the seronegative ones) and those without clinical indicators on D180. Then the remaining two cohorts (Table 2) were the discovery cohort (Cohort 3, *N* = 107) and the test cohort (Cohort 4, *N* = 20). Similar as before, proteins with a significant difference (*P*-value < 0.05) between two classes and with |log_2_(fold change)| > 0.25 in the discovery dataset were included in our final feature set. Then, some proteins (NA rate > 50%) were removed. We optimized the models’ parameters in the discovery dataset. An AUC score of 0.79 was obtained using only the PBMC proteins (Fig. 5A), indicating that PBMC proteomics had an excellent prediction ability of the antibody response after both 57 and 180 days.

The four PBMC biomarkers (PYCARD, MTMR2, PPCDC, and BRAF) selected by machine learning showed different expression patterns of the immune cells between Groups 3 and 4 (Fig. 5C). In particular, PYD and CARD domain containing (PYCARD) and phosphopantothenoylcysteine decarboxylase (PPCDC) are mainly expressed in innate immune cells, like monocytes and dendritic cells. Myotubularin related protein 2 (MTMR2) and B-Raf proto-oncogene (BRAF), on the other hand, are expressed in innate and adaptive immune cells, including NK cells, monocytes, T cells, and B cells (Fig. 5D).

However, seven participants were incorrectly predicted using this model. Specifically, participants Nos. 209 and 216 were incorrectly classified, probably because they both received simvastatin and rosuvastatin (Guerra-De-Blas et al., 2019; Karmaus et al., 2019). In addition, three participants (Nos. 212, 222, and 225) with fatty liver disease and metabolic abnormalities were also wrongly predicted, probably due to their metabolic conditions. No. 226 was misclassified may because of receiving dexamethasone and amoxicillin.

## Discussion

### Predicting the host response to CoronaVac^®^ vaccination using machine learning models

We conducted a TMT-based proteomics analysis to profile the PBMC and serum features that could affect the response to the CoronaVac^®^ vaccination. Using a set of biomarkers measured before vaccination, we built three models to predict individual NAb levels at D57 and another model to predict the persistence of NAbs until at least D180. These potential biomarkers, which were used to distinguish different host responses, were validated using an independent cohort, confirming that the changes in PBMC and serum proteins reflect the pathophysiological differences between seropositive and seronegative subjects.

#### Potential biomarkers for vaccine-induced antibody generation and persistence

The proteins used by our machine learning classifiers contain several known biomarkers for COVID-19 severity or viral infections. Of note, SERPINA10, predominantly expressed in the liver and subsequently secreted into plasma, inhibits the activity of the coagulation factors Xa and XIa in the presence of protein Z, calcium, and phospholipids (Han et al., 2000). SERPINA10 is a known discriminating feature between severe and non-severe COVID-19 (Shen et al., 2020), and can be used as a classifier of disease severity (Messner et al., 2020). Specifically, it is upregulated in the severe COVID-19 cases. In our data, SERPINA10 was downregulated in the seropositive group with a negative SHapley Additive exPlanations (SHAP) value (Fig. 2C and 2E). This result further highlights the role of coagulation during COVID-19 vaccination and indicates that SERPINA10 may contribute to reducing antibody generation. SOD3, an antioxidant enzyme, has been reported to be downregulated in the urine of severe COVID-19 cases (Bi et al., 2021). In our study, SOD3 was significantly upregulated in the seropositive group with a positive SHAP value, indicating that SOD3 may promote antibody generation. PYCARD, a key mediator of apoptosis and inflammation, is mainly involved in the innate immune response (Wang et al., 2017). It also contributes to T cell immunity stimulation and cytoskeletal rearrangements coupled to chemotaxis and antigen uptake during adaptive immunity (de Souza et al., 2021). We found PYCARD was upregulated in the seropositive group with a positive SHAP value (Fig. 5B and 5C). And thus, we suggest this protein may also promote antibody persistence. These potential biomarkers may promote or reduce antibody generation or persistence, providing therapeutic guidance for vaccination strategy.

### Mechanisms behind vaccine-induced immunity

**Figure 6.**
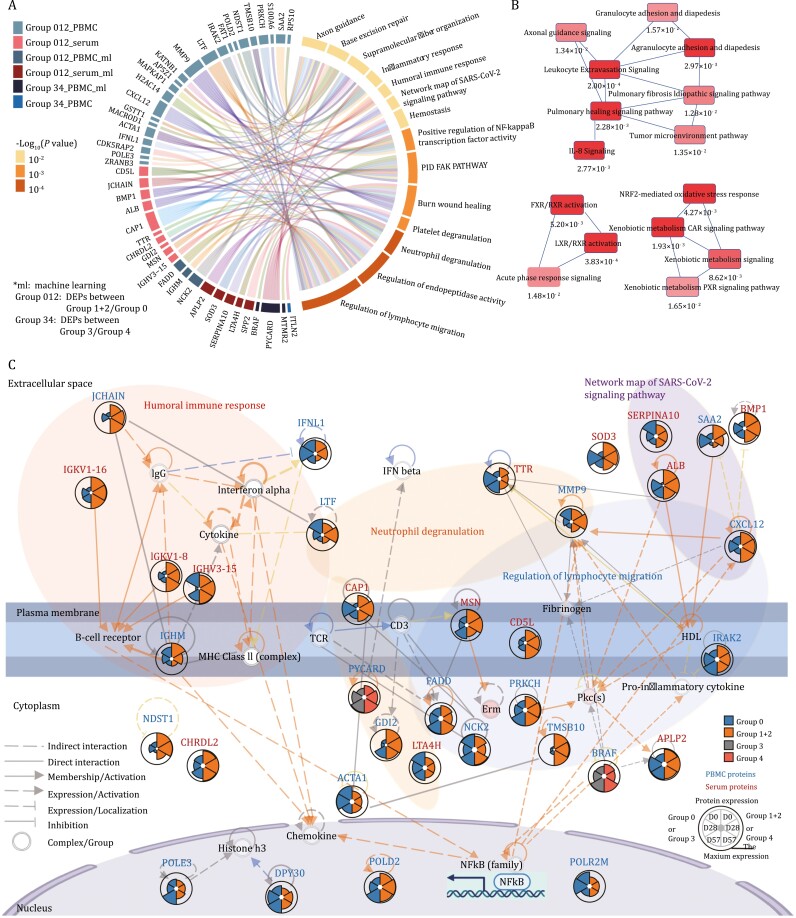
**Functional and network analyses of the seropositive and seronegative groups’ immune responses: a comparison between PBMC and serum data.** (A) Chord diagrams of the most enriched pathways based on the significantly dysregulated proteins and the potential biomarker proteins. (B) Network analysis of the most significantly enriched pathways (with their *P*-values) based on the DEPs and the potential biomarker proteins. (C) Key PBMC and serum proteins characterized in seronegative and seropositive recipients. Proteins involved in the humoral immune response, neutrophil degranulation, network maps of SARS-CoV-2 signaling pathways, and regulation of lymphocyte migration are shown in this network with their corresponding expression levels in the seronegative and the seropositive groups. Group 0: the seronegative group; Group 1 + 2: the seropositive group; Group 3: the group that became seronegative before D180; Group 4: the group that was seropositive at least until D180.

To investigate the molecular mechanisms behind vaccine-induced immunity, we integrated our proteomics analyses and thus generated a summary of the dysregulated pathways between the seropositive and the seronegative groups (Figs. 6, S6 and S7).

#### Neutrophil degranulation

In our data, we found several proteins involved in activating the neutrophil degranulation-based innate immunity, in particular, LTA4H, LTF, MMP9, TTR, CAP1, PYCARD, and GDI2. Specifically, MMP9, LTF, and CAP1 were upregulated at D28 in the seropositive group. Previous studies have shown that the release of MMP9 from neutrophils stimulates the migration of inflammatory cells and promotes inflammation and the degradation of the alveolar–capillary barrier (Davey et al., 2011). In our seropositive data, MMP9 was upregulated at D28 and then downregulated at D57 (Fig. 6C). This result suggests MMP9 may contribute to a reduced antibody generation, and is consistent with this protein being an indicator of respiratory failure (Ueland et al., 2020) and enhanced mortality risk in COVID-19 patients (C et al., 2021). Indeed, evidence has shown that neutrophil activation is a hallmark of severe SARS-CoV-2 infection (Meizlish et al., 2021). Therefore, we speculate that a modest upregulation of neutrophil degranulation may contribute to immunity activation and *vice versa*.

#### Regulation of lymphocyte migration

Most regulators of leukocyte extravasation and lymphocyte migration were elevated at D0 or during the early stages in the seropositive group: MSN, MMP9, CXCL12, FADD, NCK2, and TMSB10. In particular, MSN interacts with members of the ezrin-radixin-moesin family and regulates lymphocyte egress from lymphoid organs (Serrador et al., 1998). TMSB10, CXCL12, and NCK2 regulate the cytoskeleton organization and are involved in transmigration (Fig. 6A). By secreting proteases like MMP9, leukocytes degrade the basement membrane and penetrate the tissue interstitial spaces (Sternlicht and Werb, 2001). T cell receptors are activated through the binding by FADD and the interaction with NCK2, consistently with our immune cell analysis (Fig. 6C). Except for antigen recognition, T cell migration was positively regulated by CXCL12, FADD, and PYCARD in our seropositive groups. Intriguingly, increased FADD at baseline possibly contributed to the enhanced antibody generation at D57 (Fig. 2D). Also, higher PYCARD at baseline led to a long antibody persistence based on our machine learning models (Fig. 5C).

#### Humoral immune response

The B cell receptor is a complex of surface immunoglobulin, and some of its accessory molecules, such as IGHM, IGHV3-15, IGKV1-8, and IGKV1-16, were upregulated in our seropositive group (Fig. 6C). Following the receptor cross-linking, a complex cascade of signaling molecules results in NF-κB complex and B cell receptor activation. These IgG and cytokines are expressed by JCHAIN and LTF, which were both significantly elevated in our data after vaccination.

We clustered PBMC and serum DEPs over time (D0, D28 and D57), and grouped them into several discrete clusters using mFuzz, respectively (Fig. S6). We focused on four DEP clusters after excluding DEPs in the seronegative group (Group 0): steadily increased PBMC DEPs, decreased PBMC DEPs, increased sera DEPs, and decreased sera DEPs. Then we compared the DEPs and immune response between PBMC and serum data of the seropositive groups in Fig. S7. There were some proteins in PBMC cells that can be secreted into the serum (Fig. S7D), which were downregulated in PBMC and upregulated in serum. This cluster of PBMC and serum overlap proteins mainly functions in neutrophil degranulation and protein-lipid complex remodeling. In addition, we overlapped the proteins from these selected mFuzz clusters of PBMC and serum, and found they are involved in neutrophil degranulation, protein–lipid complex remodeling, platelet degranulation, and complement system (Figs. S6 and S7). Our data show that the neutrophil degranulation-based activation of the innate immunity, the multiple immune cell migration enhancement, and the humoral immune response activation are dysregulated at baseline and during the early stages after vaccination (Fig. 6A–C).

### Comparisons with other studies before/after vaccination

Several studies of COVID-19 vaccination have identified modulation of multiple proteins, metabolites, and gene expression after vaccination (Arunachalam et al., 2021; Liu et al., 2021; Zhang et al., 2021; Wang et al., 2022). Besides, multiple peptides and proteins have been identified to predict vaccination efficacy and differentiating COVID-19 patients from vaccinated individuals (Ma et al., 2021), which is promising for inactivated virus vaccine-related applications and translational medicine. However, no study has systematically investigated the heterogeneous hematological host responses to vaccination in both PBMCs and sera. Neither has any study presented any means to predict the host responses of vaccination before vaccination. Vaccine-induced protection against COVID-19 may involve NAbs, T cells, and innate immune mechanisms. In the comparative analysis of multiple vaccines, T cell responses in CoronaVac^®^ remains unclear (Sadarangani et al., 2021). Our PBMCs analysis showed that CD8^+^ T cells, memory B cells, and activated NK cells were increasingly upregulated in the seropositive group. Previous study showed that mRNA vaccinations can significantly enhance the innate immune response, as proven by the greater frequency CD14^+^ CD16^+^ inflammatory monocytes and higher concentration of plasma IFNγ (Arunachalam et al., 2021). In our PBMC proteome, monocytes and the IFNγ, IFNα and IFNβ signaling were elevated in the seropositive groups. A single-cell RNA-sequencing study of the PBMCs of healthy subjects revealed that, after CoronaVac^®^ vaccination, the levels of B cells, T cells, NK cells, and myeloid cells better resembled those of COVID-19 recovery controls rather than their own before vaccination (Zhang et al., 2021). Similarly, our PBMCs analysis showed that CD8^+^ T cells, memory B cells, and activated NK cells were increasingly upregulated in the seropositive group. Consistent with other reports (Wang et al., 2022), our findings support that the humoral immune response, complement activation were induced by CoronaVac^®^. What’s unique in the seropositive participants is activated regulation of lymphocyte migration pathway, which suggests enhanced immunity.

### Planning booster shot and their benefits

Due to the relatively high effectiveness of booster immunization against severe COVID-19, hospitalization, and even the Omicron variant (Xue et al., 2022), it should be strongly supported and administered at the appropriate time. The effectiveness and the safety of boosters have been assessed via large-scale randomized studies and individuals, proving the booster’s benefits and the negligible impact of its immune-mediated side effects (Zeng et al., 2022). Boosting is particularly important for specific subpopulations: individuals who generate less or shorter-lived NAbs and those who are immunocompromised, such as our seronegative participants (Group 0). Moreover, the vaccination strategy may change for recipients with heterologous or homologous vaccinations (Costa Clemens et al., 2022). Our machine learning models predict the seropositivity of individuals at D57 and their NAbs persistence until at least D180 using potential blood-derived protein biomarkers. These tools can establish which populations or individuals may generate enough and persistent NAbs, and therefore help plan precise booster administrations. Furthermore, a better balance between primary vaccination and booster may benefit more countries in the global fight against COVID-19 (Krause et al., 2021).

In summary, we performed a systematic PBMC and serum proteomic study of the heterogeneous hematological host responses to vaccination. We developed machine learning models based on panels of proteins expressed at baseline to predict antibody generation and decline after vaccination. The model can be potentially used to identify the individuals of high risk, and guide booster shot, or recommendation of other vaccines. Furthermore, our data also provides a panoramic view of the molecular changes in PBMCs and serum after vaccination.

## Limitations of the study

The findings of this study have to be considered in light of some limitations. First, the predictive models need to be further validated in larger cohorts and multicenter samples, both biologically and clinically. This model was established for CoronaVac^®^. Whether this model is applicable to other vaccines remains unclear. Nevertheless, the basic principle for different inactivated vaccines is similar. Therefore, we anticipate the findings may be of value to other inactivated vaccines. Second, the explanations for misclassifications are not very strong, may because of complex drug history. Confounding factors including age, sex, smoking history and diseases affecting immunity might influence the proteomic profiling in this study. We have analyzed some as shown in Fig. S1, and did not observe significant confounding factors, but there might be other clinical factors not included in our analysis, which could be studied in the future. Third, the B cell and T cell responses and the neutralization tests were analyzed in the mixed PBMCs but not assessed *in vitro*. Recently, the dominant strain is Omicron with evolved pathogenicity. The biological insights and predictive model established here developed may not be directly applicable to the changing viruses. However, the AI-empowered proteomic methodology established here could be directly applied to other vaccines.

## Experimental procedures

### Experimental design and statistical rationale

The overall goal was systematic investigation of host responses to COVID-19 vaccines, including the heterogeneity among the recipients, and machine learning models to predict the effectiveness of vaccination using potential biomarkers at baseline. Subject information for vaccinated recipients is summarized in Tables 1, 2 and S1. Study design of the TMT labeling-based quantitative proteomics analysis of the PBMCs and sera samples is depicted in Figs. 1A and S2A.

### Participants and samples

We recruited 163 vaccination recipients (>18 years) who were not infected with SARS-CoV-2 and some of them had stable chronic medical conditions, including hypertension, T2DM, and metabolic fatty liver disease, were eligible to be enrolled from the affiliated hospital of Hangzhou Normal University between January and February 2021, including a discovery (*N* = 137) and an independent test cohort (*N* = 26). All participants received two doses of CoronaVac^®^ (0.5 mL/dose, Sinovac life science, Beijing, China), an inactivated vaccine against SARS-CoV-2; the second dose 28 days after the first one. Blood samples were collected before vaccination (D0), then 28 (D28), 57 (D57). Blood mononuclear cells and serum were extracted from the blood samples. The xenoreactivity was also measured at D0, D28, D57 and 180 days after the first dose vaccination (D180). The NAbs for the receptor-binding domain of the SARS-CoV-2 spike protein were detected using the iFlash 2019-nCoV NAb assay (SHENZHEN YHLO BIOTECH CO., LTD, Shenzhen, China, Cat#C86109), which is a paramagnetic particle chemiluminescent immunoassay for the qualitative detection of SARS-CoV-2 NAbs in human serum and plasma using the automated iFlash immunoassay system; the cut-off value for the antibody was 10.00 AU/mL.

The participants were classified into three groups based on the xenoreactivity of their NAbs on D28 and Day 57. Specifically, Group 0 included the participants that were seronegative on D28 and D57; Group 1 included the participants that were seronegative on D28 but were seropositive on D57; Group 2 included the participants that were seropositive on D28 and D57. Groups 1 and 2 were then merged into Group 1 + 2 (all the seropositive participants). After excluding participants without clinical indicators on D180, Group 1 + 2 was then split into Group 3 (seronegative at D180) and Group 4 (seropositive at D180).

### Serum and PBMC protein extraction and digestion

From each sample, 4 µL of serum were depleted of 14 high abundant serum proteins using a human affinity depletion resin (Thermo Fisher Scientific™, San Jose, USA) and then concentrated into 50 μL through a 3K MWCO filtering unit (Thermo Fisher Scientific™, San Jose, USA). More details can be found in the manufacturer’s protocols. The resulting serum samples were then prepared for mass spectrometry as described (Shen et al., 2020). Briefly, they were denatured in 8 mol/L urea at 31.5°C for 30 min. Next, the proteins were reduced with 10 mmol/L tris (2-carboxyethyl) phosphine (TCEP) and then alkylated with 40 mmol/L iodoacetamide (IAA). Finally, the protein extracts were diluted and digested using a double step trypsinization for 16 h totally (Hualishi Tech. Ltd, Beijing, China).

PBMCs were prepared as previously described (Gao et al., 2020). Briefly, 30 μL of lysis buffer in 100 mmol/L TEAB with 20 mmol/L TCEP, and 40 mmol/L IAA were added to the PCT-Microtubes for 60 min. The proteins were digested using a mixture of trypsin and Lys-C for 120 min. Then, the digestion was arrested by adding 10% trifluoroacetic acid (TFA).

### LC-MS/MS analysis

The proteome analysis was performed similar as previously described (Shen et al., 2020). Digested peptides were cleaned-up and labeled using TMTpro 16plex label reagents (Thermo Fisher Scientific, San Jose, USA). Peptides were separated into 30 fractions, which were later combined into 15 fractions. Subsequently, the fractions were dried, redissolved in 2% ACN/0.1% formic acid. All the samples were analyzed using liquid chromatography (LC)-coupled tandem mass spectrometry (MS/MS) with a data-dependent acquisition mode on an Orbitrap 480 (Thermo Fisher Scientific, San Jose, USA). During each acquisition, peptides were analyzed using a 30 min-long LC gradient (from 7 to 30% buffer B). The m/z range of MS1 was 375–1,800, with a resolution of 60,000, normalized Automatic Gain Control (AGC) target of 300%, maximum ion injection time (max IT) of 50 ms, and compensation voltages of −48 V and −68 V for FAIMS Pro™. MS/MS experiments were performed with a resolution of 30,000, normalized AGC target of 200%, and 86 ms max IT for Serum and 100 ms for PBMC. The turbo-TMT and the advanced peak determination were enabled.

### Database search for proteomics quantification

The mass spectrometric data were analyzed using Proteome Discoverer (Version 2.4.0.305, Thermo Fisher Scientific) and the *Homo sapiens* protein database downloaded from UniProtKB on 27 April 2020 (Fasta file containing 20,301 reviewed protein sequences). The database search was performed as previously described (Shen et al., 2020), including Carbamidomethyl (C) as a fixed modification and oxidation (M) as a variable modification. The false discovery rate (FDR) was set as 0.01. Data normalization was performed against the total peptide amount. Other parameters followed the default setup.

### Quality control of the proteome data

The quality of the proteomics data was ensured at multiple levels. A pool of samples labeled by TMTpro-134N was used as the control for aligning the data from different batches. Also, we assessed the reproducibility of the data using technical replicates, water samples (buffer A) as blanks every four injections to avoid carry-over.

After removing the proteins with over 90% missing values, 6331 proteins of PBMC and 961 of serum underwent quality controls. We then assessed the coefficient of variation in the pooled samples (Fig. S2B). Finally, the Pearson’s correlation values of the technical replicates (17 PBMC samples and three serum samples) were used to evaluate the reproducibility of the data (Fig. S2C).

### Statistical analysis of clinical indicators

Continuous variables were calculated by Student’s *t*-test or Welch’s *t*-test, Pearson *χ*^2^ test or Fisher’s exact test for the analysis of categorical outcomes. We calculated Geometric Mean Titers (GMT) of the neutralizing antibody titers and the overall anti-Spike IgG levels, using the *t*-test method to compare the difference. Statistical analysis was performed by IBM SPSS Statistics 26 (Armonk, NY: IBM Corp).

### Differential expression analysis

A set of statistical tools were used to process and analyze our proteomics data. First, the batch effect of the serum proteome was removed using the R package combat. No other significant batch effect was highlighted by principal component analysis (Fig. S2D and S2E). For comparing the protein expressions between groups, the log_2_(fold change) was calculated using the mean values of each group. A two-sided unpaired Welch’s *t*-test was performed for each group pair. A one-way analysis of variance (ANOVA) was performed among three groups at three time points. Finally, the adjusted *P*-values were calculated using the B-H correction.

DEPs were selected by imposing the B-H adjusted *P*-values to be less than 0.05 and the absolute log_2_(fold change) larger than 0.25. Next, a soft clustering of the time series data was performed using MFuzz (version 2.48.0). We clustered the PBMC and serum DEPs expression along time using default settings (Fig. S6). The single-cell RNA expression of PBMCs was derived from the Human Proteins Atlas (Karlsson et al., 2021).

### Estimation of the immune cell type fractions

CIBERSORT is an analytical tool for estimating the cell composition of tissues using their gene expression profiles (Newman et al., 2015). In CIBERSORT, the relative amounts of 20 human immune cell types (including naïve and memory B cells, seven T cell types, NK cells, plasma cells, monocytes, etc.) were estimated in our PBMC bulk cells using the leukocyte gene signature matrix. In addition, vaccinated individuals were divided into seronegative and seropositive groups, and the fraction of each immune cell type was investigated and visualized with bar plots using R software (R 4.0.5).

### Machine learning

For prediction of NAbs generation on D57, we used the samples from a discovery cohort (Cohort 1, *N* = 137) to optimize the model’s parameters, the discovery dataset was randomly split into a training (80%) and a validation (20%) dataset. To establish the features for our machine learning models, we used a differential protein expression analysis which returned a set of biomarkers from the PBMCs and the serum (Figs. 2A and 5A). Proteins with a significant difference (*P*-value < 0.05) between two classes and with |log_2_(fold change)| > 0.25 in the training dataset were included in our final feature set. Then, sparse proteins (NA rate > 50%) were removed. The missing values were imputed with the minimum of each protein. We decided on the top *N* best features as the final feature set for our model, as well as the optimal parameters, by searching for the highest AUC in the validation dataset. All individual models for these two tasks can achieve an AUC of 1.0 in the validation dataset. Finally, the results illustrated in this paper were derived from the model with the best features and parameters. The implementation of machine learning was done using Python 3.8.10 and xgboost 1.4.2 python package (Chen and Guestrin, 2016).The model was then tested using an independent test cohort (Cohort 2, *N* = 26): the first based on PBMC biomarkers and the second on serum biomarkers. We next developed a third model that was an ensemble of the two previous ones. This third model led to an AUC of 0.87, which was higher than using PBMC or serum proteins individually.

For prediction of NAbs persistence till D180, we discarded participants from Group 0 (the seronegative ones) and those without clinical indicators on D180, and the remaining two cohorts: a training cohort (Cohort 3, *N* = 107) and a test cohort for the validation (Cohort 4, *N* = 20). We optimized the models’ parameters in the training and a validation dataset. Similarly, we tested in Cohort 4, and an AUC score of 0.79 was obtained using only the PBMC proteins (Fig. 5A).

### Functional analyses

Specifically, we investigated 38 PBMC DEPs and 14 serum DEPs from different immune response groups using a two-sided unpaired Welch’s *t*-test, and 985 DEPs from PBMCs and 129 DEPs from serum were evaluated using the ANOVA test, biomarker proteins were also included. Several pathway analysis tools were used to perform the functional analysis of our significantly DEPs. Enrichments analyses based on Gene Ontology processes, KEGG pathways, Reactome gene sets, and Wiki pathways were performed using the web-based platform of Metascape (Zhou et al., 2019). With an Ingenuity pathway analysis (Kramer et al., 2014) of the dysregulated proteins, we identified the most significantly dysregulated pathways; *P*-values were based on a right-tailed Fisher’s Exact Test, and the enriched pathways’ overall activation/inhibition state was predicted using the *z*-score. A pathway’s regulation was significant if its *P*-value < 0.05. Gene Set Variation Analysis (GSVA) was performed using the R package GSVA (version 3.11) (Hanzelmann et al., 2013) to identify the most dysregulated pathways (Canonical pathways) between the seronegative and the seropositive groups (B-H adjusted *P*-value < 0.05). The functional network images generated by Metascape were visualized with Cytoscape (version 3.9.0) to generate the network of predicted associations for a specific group of proteins (Otasek et al., 2019).

## Supplementary Material

pwad004_suppl_Supplementary_Materials

pwad004_suppl_Supplementary_Table_S1

pwad004_suppl_Supplementary_Table_S2

pwad004_suppl_Supplementary_Table_S3

pwad004_suppl_Supplementary_Table_S4

pwad004_suppl_Supplementary_Table_S5

pwad004_suppl_Supplementary_Table_S6

## Data Availability

All mass spectrometry data in this paper are available in the platform iProX (Project ID: IPX0004305000).

## Abbreviations

AGC: automatic gain control
AUC: area under curve
B-H: Benjamini-Hochberg
COVID-19: coronavirus disease 2019
CV: coefficients of variance
DEPs: differentially expressed proteins
ECM: extracellular matrix
FDR: false discovery rate
GMT: Geometric Mean Titers; GSVA, gene-set variation analysis
IFN: interferon
MAFLD: metabolic associated fatty liver disease
PBMCs: peripheral blood mononuclear cells
SARS-Cov-2: severe acute respiratory syndrome coronavirus 2
SHAP: SHapley Additive exPlanations
T2DM: type 2 diabetes mellitus

## Code availability

All the codes needed to reproduce the empirical results reported in this paper are available in an open science data repository GitHub at/guomics-lab/CVDHZ.

## References

[CIT0001] Ai J , Guo J, Zhang H et al. Cellular basis of enhanced humoral immunity to SARS-CoV-2 upon homologous or heterologous booster vaccination analyzed by single-cell immune profiling. Cell Discov 2022;8:114. 36270988 10.1038/s41421-022-00480-5 PMC9587260

[CIT0002] Arunachalam PS , Scott MKD, Hagan T et al. Systems vaccinology of the BNT162b2 mRNA vaccine in humans. Nature 2021;596:410–416. 34252919 10.1038/s41586-021-03791-x PMC8761119

[CIT0003] Bi X , Liu W, Ding X et al. Proteomic and metabolomic profiling of urine uncovers immune responses in patients with COVID-19. Cell Rep 2021;38:110271. 35026155 10.1016/j.celrep.2021.110271 PMC8712267

[CIT0004] Botham KM , Wheeler-Jones CP. Postprandial lipoproteins and the molecular regulation of vascular homeostasis. Prog Lipid Res 2013;52:446–464. 23774609 10.1016/j.plipres.2013.06.001

[CIT0005] Brannagan TH 3rd , Auer-Grumbach M, Berk JL et al. ATTR amyloidosis during the COVID-19 pandemic: insights from a global medical roundtable. Orphanet J Rare Dis 2021;16:204. 33957949 10.1186/s13023-021-01834-0 PMC8100737

[CIT0006] C DA-M , Couto AES, Campos LCB et al. MMP-2 and MMP-9 levels in plasma are altered and associated with mortality in COVID-19 patients. Biomed Pharmacother 2021;142:112067. 34449310 10.1016/j.biopha.2021.112067 PMC8376652

[CIT0007] Chen TQ , Guestrin C. XGBoost: a scalable tree boosting system. Kdd’16: Proceedings of the 22nd Acm Sigkdd International Conference on Knowledge Discovery and Data Mining, 2016, 785–794.

[CIT0008] Chen Y , Yin S, Tong X et al. Dynamic SARS-CoV-2-specific B-cell and T-cell responses following immunization with an inactivated COVID-19 vaccine. Clin Microbiol Infect 2022;28:410–418. 34715346 10.1016/j.cmi.2021.10.006 PMC8547974

[CIT0009] Costa Clemens SA , Weckx L, Clemens R et al. Heterologous versus homologous COVID-19 booster vaccination in previous recipients of two doses of CoronaVac COVID-19 vaccine in Brazil (RHH-001): a phase 4, non-inferiority, single blind, randomised study. Lancet 2022;399:521–529. 35074136 10.1016/S0140-6736(22)00094-0 PMC8782575

[CIT0010] Davey A , McAuley DF, O’Kane CM. Matrix metalloproteinases in acute lung injury: mediators of injury and drivers of repair. Eur Respir J 2011;38:959–970. 21565917 10.1183/09031936.00032111

[CIT0011] de Souza JG , Starobinas N, Ibanez OCM. Unknown/enigmatic functions of extracellular ASC. Immunology 2021;163:377–388. 34042182 10.1111/imm.13375 PMC8274145

[CIT0012] Falsey AR , Frenck RW Jr, Walsh EE et al. SARS-CoV-2 neutralization with BNT162b2 vaccine dose 3. N Engl J Med 2021;385:1627–1629. 34525276 10.1056/NEJMc2113468 PMC8461567

[CIT0013] Gao H , Zhang F, Liang S et al. Accelerated lysis and proteolytic digestion of biopsy-level fresh-frozen and FFPE tissue samples using pressure cycling technology. J Proteome Res 2020;19:1982–1990. 32182071 10.1021/acs.jproteome.9b00790

[CIT0014] Guerra-De-Blas PDC , Bobadilla-Del-Valle M, Sada-Ovalle I et al. Simvastatin enhances the immune response against mycobacterium tuberculosis. Front Microbiol 2019;10:2097. 31616387 10.3389/fmicb.2019.02097 PMC6764081

[CIT0015] Han X , Fiehler R, Broze GJ Jr. Characterization of the protein Z-dependent protease inhibitor. Blood 2000;96:3049–3055. 11049983

[CIT0016] Hanzelmann S , Castelo R, Guinney J. GSVA: gene set variation analysis for microarray and RNA-seq data. BMC Bioinf 2013;14:7. 10.1186/1471-2105-14-7 PMC3618321 23323831

[CIT0017] Iizuka Y , Cichocki F, Sieben A et al. UNC-45A Is a nonmuscle myosin IIA chaperone required for NK cell cytotoxicity via control of lytic granule secretion. J Immunol 2015;195:4760–4770. 26438524 10.4049/jimmunol.1500979 PMC5189640

[CIT0018] Kabra NH , Kang C, Hsing LC et al. T cell-specific FADD-deficient mice: FADD is required for early T cell development. Proc Natl Acad Sci USA 2001;98:6307–6312. 11353862 10.1073/pnas.111158698 PMC33464

[CIT0019] Karlsson M , Zhang C, Mear L et al. A single-cell type transcriptomics map of human tissues. Sci Adv 2021;7:eabh2169. 34321199 10.1126/sciadv.abh2169 PMC8318366

[CIT0020] Karmaus PW , Shi M, Perl S et al. Effects of rosuvastatin on the immune system in healthy volunteers with normal serum cholesterol. JCI Insight 2019;4:e131530. 31573980 10.1172/jci.insight.131530 PMC6948773

[CIT0021] Kramer A , Green J, Pollard J et al. Causal analysis approaches in ingenuity pathway analysis. Bioinformatics 2014;30:523–530. 24336805 10.1093/bioinformatics/btt703 PMC3928520

[CIT0022] Krause PR , Fleming TR, Peto R et al. Considerations in boosting COVID-19 vaccine immune responses. Lancet 2021;398:1377–1380. 34534516 10.1016/S0140-6736(21)02046-8 PMC8437678

[CIT0023] Lee A , Wong SY, Chai LYA et al. Efficacy of COVID-19 vaccines in immunocompromised patients: systematic review and meta-analysis. BMJ 2022;376:e068632. 35236664 10.1136/bmj-2021-068632 PMC8889026

[CIT0024] Liu J , Wang J, Xu J et al. Comprehensive investigations revealed consistent pathophysiological alterations after vaccination with COVID-19 vaccines. Cell Discov 2021;7:99. 34697287 10.1038/s41421-021-00329-3 PMC8546144

[CIT0025] Ma ML , Shi DW, Li Y et al. Systematic profiling of SARS-CoV-2-specific IgG responses elicited by an inactivated virus vaccine identifies peptides and proteins for predicting vaccination efficacy. Cell Discov 2021;7:67. 34400612 10.1038/s41421-021-00309-7 PMC8367966

[CIT0026] Meizlish ML , Pine AB, Bishai JD et al. A neutrophil activation signature predicts critical illness and mortality in COVID-19. Blood Adv 2021;5:1164–1177. 33635335 10.1182/bloodadvances.2020003568 PMC7908851

[CIT0027] Messner CB , Demichev V, Wendisch D et al. Ultra-high-throughput clinical proteomics reveals classifiers of COVID-19 infection. Cell Syst 2020;11:11–24.e4. 32619549 10.1016/j.cels.2020.05.012 PMC7264033

[CIT0028] Newman AM , Liu CL, Green MR et al. Robust enumeration of cell subsets from tissue expression profiles. Nat Methods 2015;12:453–457. 25822800 10.1038/nmeth.3337 PMC4739640

[CIT0029] Otasek D , Morris JH, Boucas J et al. Cytoscape automation: empowering workflow-based network analysis. Genome Biol 2019;20:185. 31477170 10.1186/s13059-019-1758-4 PMC6717989

[CIT0030] Sadarangani M , Marchant A, Kollmann TR. Immunological mechanisms of vaccine-induced protection against COVID-19 in humans. Nat Rev Immunol 2021;21:475–484. 34211186 10.1038/s41577-021-00578-z PMC8246128

[CIT0031] Schroeder HW Jr , Cavacini L. Structure and function of immunoglobulins. J Allergy Clin Immunol 2010;125:S41–S52. 20176268 10.1016/j.jaci.2009.09.046 PMC3670108

[CIT0032] Serrador JM , Nieto M, Alonso-Lebrero JL et al. CD43 interacts with moesin and ezrin and regulates its redistribution to the uropods of T lymphocytes at the cell-cell contacts. Blood 1998;91:4632–4644. 9616160

[CIT0033] Sette A , Crotty S. Adaptive immunity to SARS-CoV-2 and COVID-19. Cell 2021;184:861–880. 33497610 10.1016/j.cell.2021.01.007 PMC7803150

[CIT0034] Shen B , Yi X, Sun Y et al. Proteomic and metabolomic characterization of COVID-19 patient sera. Cell 2020;182:59–72.e15. 32492406 10.1016/j.cell.2020.05.032 PMC7254001

[CIT0035] Sternlicht MD , Werb Z. How matrix metalloproteinases regulate cell behavior. Annu Rev Cell Dev Biol 2001;17:463–516. 11687497 10.1146/annurev.cellbio.17.1.463 PMC2792593

[CIT0036] Ueland T , Holter JC, Holten AR et al. Distinct and early increase in circulating MMP-9 in COVID-19 patients with respiratory failure. J Infect 2020;81:e41–e43. 32603675 10.1016/j.jinf.2020.06.061 PMC7320854

[CIT0037] van der Graaf R , Browne JL, Baidjoe AY. Vaccine equity: past, present and future. Cell Rep Med 2022;3:100551. 35474741 10.1016/j.xcrm.2022.100551 PMC8831140

[CIT0038] Wang Y , Ning X, Gao P et al. Inflammasome activation triggers caspase-1-mediated cleavage of cGAS to regulate responses to DNA virus infection. Immunity 2017;46:393–404. 28314590 10.1016/j.immuni.2017.02.011

[CIT0039] Wang Y , Wang X, Luu LDW et al. Proteomic and metabolomic signatures associated with the immune response in healthy individuals immunized with an inactivated SARS-CoV-2 vaccine. Front Immunol 2022;13:848961. 35686122 10.3389/fimmu.2022.848961 PMC9171821

[CIT0040] Xue J-B , Lai D-Y, Jiang H-W et al. Landscape of the RBD-specific IgG, IgM, and IgA responses triggered by the inactivated virus vaccine against the Omicron variant. Cell Discov 2022;8:15. 35169145 10.1038/s41421-022-00380-8 PMC8847627

[CIT0041] Zeng G , Wu Q, Pan H et al. Immunogenicity and safety of a third dose of CoronaVac, and immune persistence of a two-dose schedule, in healthy adults: interim results from two single-centre, double-blind, randomised, placebo-controlled phase 2 clinical trials. Lancet Infect Dis 2022;22:483–495. 34890537 10.1016/S1473-3099(21)00681-2 PMC8651254

[CIT0042] Zhang H , Hu Y, Jiang Z et al. Single-cell sequencing and immune function assays of peripheral blood samples demonstrate positive responses of an inactivated SARS-CoV-2 vaccine. SSRN. 2021. doi:10.2139/ssrn.3774153.

[CIT0043] Zhao X , Li D, Ruan W et al. Effects of a prolonged booster interval on neutralization of omicron variant. N Engl J Med 2022;386:894–896. 35081296 10.1056/NEJMc2119426 PMC8809506

[CIT0044] Zhou Y , Zhou B, Pache L et al. Metascape provides a biologist-oriented resource for the analysis of systems-level datasets. Nat Commun 2019;10:1523. 30944313 10.1038/s41467-019-09234-6 PMC6447622

