# Supplementary material for: Longitudinal proteomic investigation of COVID-19 vaccination
Source: Protein Cell. 2023 Feb 6;14(9):668–82. doi: 10.1093/procel/pwad004 (PMC10501184; doi:10.1093/procel/pwad004)
Supplement: pwad004_suppl_Supplementary_Materials [file pwad004_suppl_supplementary_materials.pdf]

Fig. S1. Factors associated with seropositivity of neutralizing antibodies at D57 and D180, respectively. (A) Multivariable logistic regression analysis was used to investigate factors associated with the generation of NAbs at D57 after adjusting sex, BMI and diastolic bold pressure. **(B)** Multivariable logistic regression analysis was used to investigate factors associated with the persistence of NAbs at D180 after adjusting leukocytes, monocytes, RBC, lymphocytes, hemoglobin, LDL, HDL, TG, ALT, and AST.

Fig. S2. Quality controls of the proteomics data. (A) Study design of our TMT-labeling-based quantitative proteomics analysis of PBMCs and sera samples. Together, PBMCs and serum generated 528 peptide samples, including 33 pooled controls, distributed into 33 batches and analyzed using TMTpro 16-plex labeling based proteomics. **(B)** The proteomics data's coefficients of variation were calculated using the abundance of the quantified proteins in the 33 pooled controls of the 33 batches. Also, they were computed after removing the outliers. **(C)** Quality control of the technical replicates based on the Pearson correlation coefficients. **(D-E)** PCA analyses were performed on all the proteomics data derived from the PBMC **(D)** and serum samples **(E)**, for three immune response groups on D57 (Group 0, Group 1, and Group 2) and two immune response groups on D180 (Group 3 and Group 4). Group 1: the late seropositive group; Group 2: the early seropositive group.

Fig. S3. Machine learning-based prediction of Group 1 (being negative at D28 and then converting) and Group 0 (being negative at D28 and never converting) before vaccination. (A) Our machine learning-based predictor was based on PBMC, serum, and both types of proteins. We used the samples from a discovery cohort (Cohort 1, N = 137) to optimize the model's parameters. The model was then tested using a test cohort (Cohort 2, N = 26): the first based on PBMC biomarkers and the second on serum biomarkers. We next developed a third model that was an ensemble of the two previous ones. This third model led to an AUC of 0.853, which was higher than using PBMC or serum proteins individually. **(B)** The SHAP values of the five PBMC proteins were prioritized using the machine learning model. **(C)** The SHAP values of the five serum proteins were prioritized using the machine learning model. Identification of NAb status-associated proteins in PBMC **(D)** and serum **(E)** using volcano plot analysis on D0, D28, and D57 (two-sided unpaired Welch's *t*-test). The $\log_{10}$ (B-H adjusted *P*-value) is plotted as a function of the $\log_2$(fold change) between Group 1 and Group 0 samples (B-H adjusted *P*-value < 0.05, $|\log_2$(fold change)$| > 0.25$).

Fig. S4. Immune response and pathway analyses using the PBMC data. (A) Heatmap of 985 proteins selected using the ANOVA test. Each protein is compared among the three immune response groups (Group 0, 1, and 2) on D0, D28, and D57 (*P*-value < 0.05). Group 0: the seronegative group; Group 1: the late seropositive group; Group 2: the early seropositive group. **(B-D)** Pathways' enrichment based on the proteins selected using the ANOVA test to compare the three immune response groups on D0 **(B)**, D28 **(C)**, and D57 **(D)** using Metascape ($\log_{10}$ *P*-value). **(E)**

Barplots visualizing the estimated proportions of 20 immune cell types in each PBMC sample. Each column represents a sample; the colors indicate the inferred immune cell components. **(F)** Average proportions of 20 immune cell types, in Group 0, Group 1, and Group 2, at three time points. The asterisks indicate the statistical significance based on the Kruskal-Wallis test. *P*-value: *, < 0.05; **, < 0.01; ***, < 0.001. **(G)** Barplots visualizing the estimated proportions of 20 immune cell types, in Group 0, Group 1, and Group 2, at three time points. Different colors indicate the predicted composition of immune cell types.

Fig. S5. Immune response and pathway analysis using the serum data. (A) Heatmap of 129 proteins selected using the ANOVA test. Each protein is compared between the three immune response groups (Group 0, 1, and 2) on D0, D28, and D57 (*P*-value < 0.05). Group 0: the seronegative group; Group 1: the late seropositive group; Group 2: the early seropositive group. **(B)** Pathways' enrichment based on the proteins selected in **(A)** using Metascape. **(C-D)** Comparison among three immune response groups (Group 0, 1, and 2) of the canonical pathways of proteins in (A) by IPA.

Fig. S6. Immune response and pathway analysis using the PBMC and the serum data. (A-B) Pathways' enrichment analysis of selected PBMC proteins. Specifically, 6331 PBMC proteins and 961 serum proteins were grouped into 12 discrete clusters using mFuzz, respectively. This analysis included all the proteins from the PBMC dataset that steadily increased **(A)** or decreased **(B)** over time. And the serum dataset that steadily increased **(C)** or decreased **(D)** over time. The 20 most significantly enriched pathways involving these DEPs were analyzed using Metascape.

Fig. S7. Immune response and network analysis of the seropositive groups: comparison between PBMC and serum data. The most significantly dysregulated proteins identified using mFuzz from the following groups are here compared: **(A)** upregulated in both PBMC and serum, **(B)** downregulated in both PBMC and serum, **(C)** upregulated in PBMC and downregulated in serum, and **(D)** downregulated in PBMC and upregulated in serum. The Venn diagrams show the overlaps between the PBMC (blue) and the serum proteins (red). Pathways and heatmaps were generated from the overlapping proteins from each pair. **(E)** The most significantly enriched networks generated using the DEPs from **(A-D)**. The proteins involved in the complement system, including platelet degranulation, neutrophil degranulation, and protein-lipid complex remodeling.

Figure S1

A

Seropositive of NAb titers at D57

Univariable analysis

	pvalue	Odd ratio (95%CI)
Age	0.005	0.930(0.884−0.979)
Sex (vs male)	0.042	0.364(0.137−0.963)
BMI	0.027	0.878(0.782−0.985)
Diastolic Blood Pressure	0.030	0.951(0.909−0.995)
Systolic Blood Pressure	0.093	0.970(0.937−1.005)
NAb titers at day 28	<0.001	3.173(1.755−5.737)
Leukocytes	0.757	0.950(0.686−1.315)
Monocytes	0.370	5.804(0.124−271.388)
RBC	0.428	0.698(0.287−1.698)
Lymphocytes	0.854	0.927(0.413−2.081)
Plateles	0.377	1.004(0.996−1.012)
Neutrophils	0.652	0.908(0.596−1.383)
Hemoglobin	0.693	0.995(0.969−1.021)
UA	0.444	0.998(0.993−1.003)
GGT	0.340	1.017(0.983−1.052)
TBIL	0.986	1.001(0.930−1.077)
LDL	0.248	1.457(0.769−2.760)
HDL	0.267	2.542(0.489−13.217)
TC	0.180	1.453(0.841−2.509)
TG	0.914	0.956(0.424−2.157)
Glucose	0.847	1.047(0.655−1.674)
IR	0.262	0.896(0.741−1.085)
Alb	0.119	1.179(0.958−1.449)
CRP	0.922	1.007(0.868−1.169)
Cr	0.865	0.999(0.983−1.014)
ALT	0.538	1.011(0.977−1.046)
AST	0.713	1.011(0.952−1.074)
Drug(vs without drug)	0.688	0.729(0.156−3.400)
MAFLD(vs non-MAFLD)	0.099	2.261(0.857−5.968)
Hypertension(vs non-hypertension)	0.553	1.500(0.393−5.718)
T2DM (vs non-T2DM)	0.562	1.944(0.206−18.367)

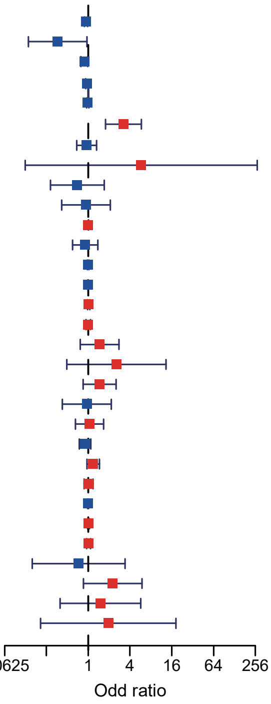

Multivariable analysis

	pvalue	Odd ratio (95%CI)
Age	0.136	0.956(0.902−1.014)
Sex (vs male)	0.992	0.994(0.294−3.357)
BMI	0.053	0.855(0.729−1.002)
Diastolic Blood Pressure	0.467	0.977(0.918−1.040)
NAb titers at day 28	<0.001	3.079(1.663−5.698)

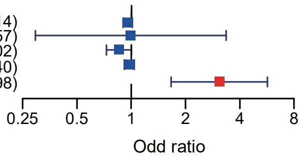

B

Seropositive of NAb titers at D180

Univariable analysis

	pvalue	Odd ratio (95%CI)
Age	0.340	0.984(0.950−1.018)
Sex (vs male)	0.061	2.017(0.967−4.208)
BMI	0.699	0.981(0.890−1.081)
Diastolic Blood Pressure	0.634	1.008(0.975−1.043)
Systolic Blood Pressure	0.209	1.018(0.990−1.046)
NAb titers at day 28	0.003	1.121(1.041−1.207)
NAb titers at day 57	0.002	1.030(1.011−1.049)
Leukocytes	0.028	1.334(1.032−1.725)
Monocytes	0.043	16.744(1.100−254.957)
RBC	0.001	3.425(1.618−7.250)
Lymphocytes	0.003	2.748(1.394−5.416)
Platelets	0.060	1.006(1.000−1.013)
Neutrophils	0.390	1.150(0.836−1.580)
Hemoglobin	0.029	1.025(1.002−1.048)
UA	0.087	1.003(1.000−1.007)
GGT	0.089	1.014(0.998−1.031)
TBIL	0.062	0.943(0.888−1.003)
LDL	0.047	1.593(1.007−2.522)
HDL	0.041	0.267(0.075−0.950)
TC	0.036	1.525(1.028−2.261)
TG	0.001	3.010(1.603−5.650)
Glucose	0.449	0.864(0.592−1.261)
IR	0.240	1.115(0.930−1.336)
Alb	0.061	1.156(0.993−1.346)
CRP	0.846	0.983(0.828−1.168)
Cr	0.610	1.003(0.991−1.015)
ALT	0.025	1.024(1.003−1.045)
AST	0.021	1.051(1.007−1.097)
Drug(vs without drug)	0.069	0.422(0.166−1.070)
MAFLD(vs non-MAFLD)	0.084	0.521(0.249−1.092)
Hypertension(vs non-hypertension)	0.181	0.508(0.188−1.370)
T2DM (vs non-T2DM)	0.656	1.657(0.180−15.273)

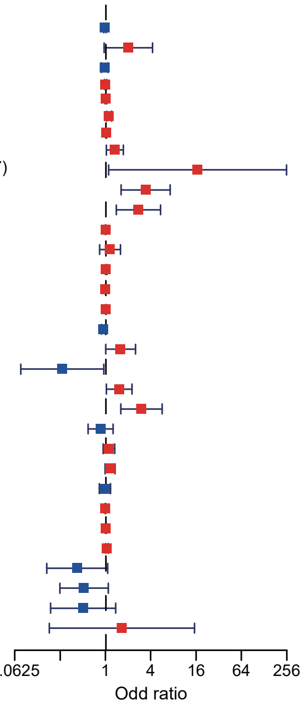

Multivariable analysis

	pvalue	Odd ratio (95%CI)
NAb titers at day 28	0.026	1.103(1.012−1.202)
NAb titers at day 57	0.047	1.020(1.000−1.040)
Lukocytes	0.418	0.835(0.539−1.292)
Monocytes	0.240	3.726(0.415−33.487)
RBC	0.087	2.554(0.874−7.467)
Lymphocytes	0.075	2.493(0.910−6.828)
LDL	0.378	0(0−9.37E+37)
HDL	0.379	0(0−1.15E+38)
TC	0.377	1.34E+31(0−1.34E+100)
TG	0.386	0(0−3.36E+17)
ALT	0.689	0.989(0.937−1.044)
AST	0.664	1.024(0.920−1.140)

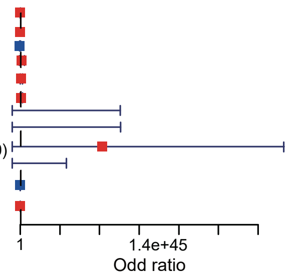

Figure S2

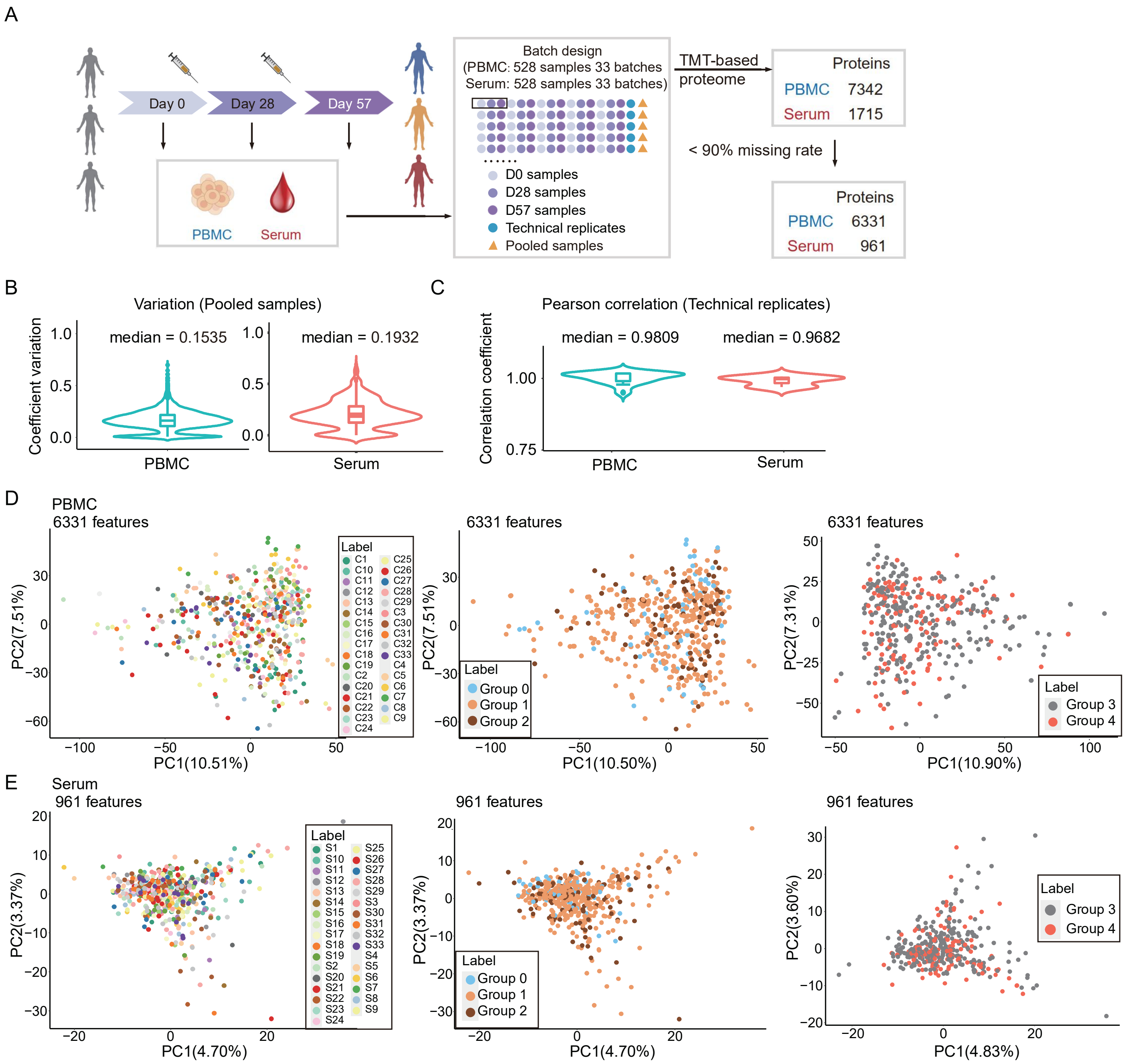
A
Day 0
Day 28
Day 57
PBMC
Serum
Batch design
(PBMC: 528 samples 33 batches
Serum: 528 samples 33 batches)
D0 samples
D28 samples
D57 samples
Technical replicates
Pooled samples
TMT-based proteome
Proteins
PBMC 7342
Serum 1715
< 90% missing rate
Proteins
PBMC 6331
Serum 961
B
Variation (Pooled samples)
Coefficient variation
median = 0.1535
median = 0.1932
PBMC
Serum
C
Pearson correlation (Technical replicates)
Correlation coefficient
median = 0.9809
median = 0.9682
PBMC
Serum
D
PBMC
6331 features
PC1(10.51%)
PC2(7.51%)
PC1(10.50%)
PC2(7.51%)
Group 0
Group 1
Group 2
PC1(10.90%)
PC2(7.31%)
Group 3
Group 4
Label
E
Serum
961 features
PC1(4.70%)
PC2(3.37%)
PC1(4.83%)
PC2(3.60%)

Figure S3

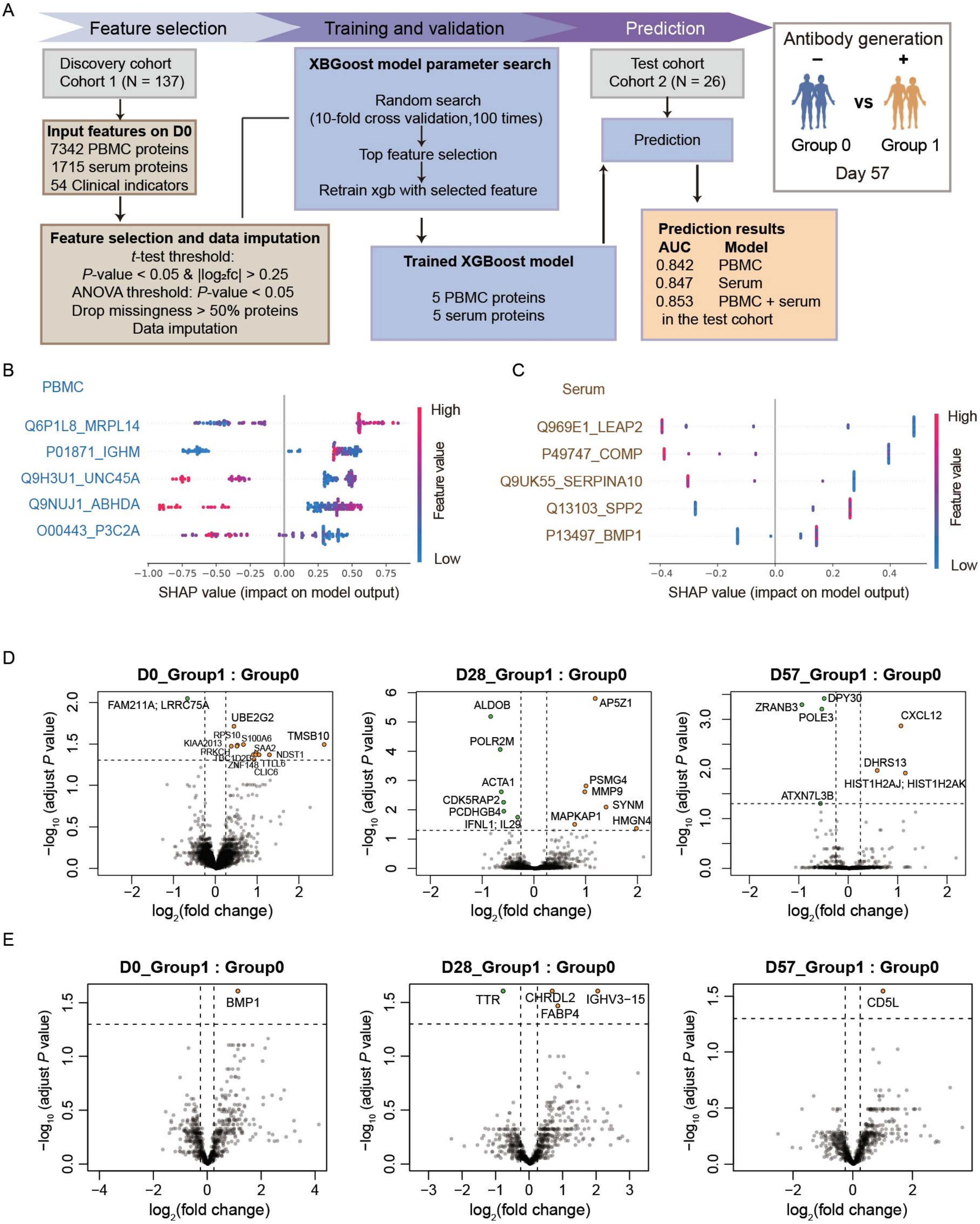
A
Feature selection
Training and validation
Prediction
Discovery cohort
Cohort 1 (N = 137)
Input features on D0
7342 PBMC proteins
1715 serum proteins
54 Clinical indicators
Feature selection and data imputation
t-test threshold:
P-value < 0.05 & |log2fc| > 0.25
ANOVA threshold: P-value < 0.05
Drop missingness > 50% proteins
Data imputation
XBGoost model parameter search
Random search
(10-fold cross validation,100 times)
Top feature selection
Retrain xgb with selected feature
Trained XGBoost model
5 PBMC proteins
5 serum proteins
Test cohort
Cohort 2 (N = 26)
Prediction
Prediction results
AUC Model
0.842 PBMC
0.847 Serum
0.853 PBMC + serum
in the test cohort
Antibody generation
− +
vs
Group 0 Group 1
Day 57
B
PBMC
Q6P1L8_MRPL14
P01871_IGHM
Q9H3U1_UNC45A
Q9NUJ1_ABHDA
O00443_P3C2A
High
Feature value
Low
SHAP value (impact on model output)
C
Serum
Q969E1_LEAP2
P49747_COMP
Q9UK55_SERPINA10
Q13103_SPP2
P13497_BMP1
High
Feature value
Low
SHAP value (impact on model output)
D
D0_Group1 : Group0
FAM211A; LRRC75A
UBE2G2
TMSB10
D28_Group1 : Group0
ALDOB
AP5Z1
POLR2M
ACTA1
CDK5RAP2
PCDHGB4
IFNL1; IL29
PSMG4
MMP9
SYNM
MAPKAP1
HMGN4
D57_Group1 : Group0
ZRANB3
DPY30
POLE3
CXCL12
DHRS13
HIST1H2AJ; HIST1H2AK
ATXN7L3B
−log10 (adjust P value)
log2(fold change)
E
D0_Group1 : Group0
BMP1
D28_Group1 : Group0
TTR
CHRDL2
IGHV3−15
FABP4
D57_Group1 : Group0
CD5L
−log10 (adjust P value)
log2(fold change)

Figure S4

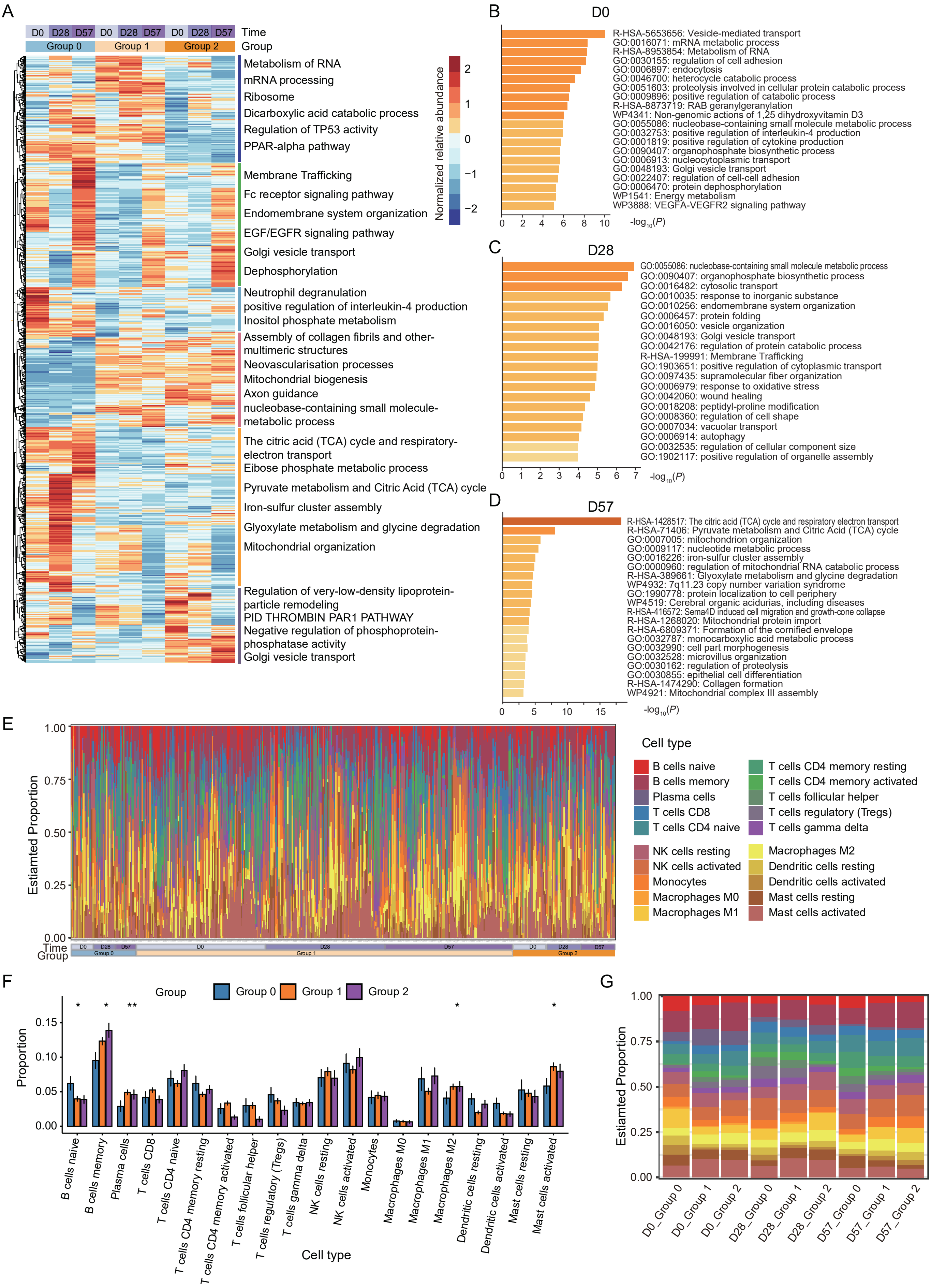

A
D0 D28 D57 D0 D28 D57 D0 D28 D57 Time
Group 0 Group 1 Group 2 Group
Metabolism of RNA
mRNA processing
Ribosome
Dicarboxylic acid catabolic process
Regulation of TP53 activity
PPAR-alpha pathway
Membrane Trafficking
Fc receptor signaling pathway
Endomembrane system organization
EGF/EGFR signaling pathway
Golgi vesicle transport
Dephosphorylation
Neutrophil degranulation
positive regulation of interleukin-4 production
Inositol phosphate metabolism
Assembly of collagen fibrils and other-multimeric structures
Neovascularisation processes
Mitochondrial biogenesis
Axon guidance
nucleobase-containing small molecule-metabolic process
The citric acid (TCA) cycle and respiratory-electron transport
Eibose phosphate metabolic process
Pyruvate metabolism and Citric Acid (TCA) cycle
Iron-sulfur cluster assembly
Glyoxylate metabolism and glycine degradation
Mitochondrial organization
Regulation of very-low-density lipoprotein-particle remodeling
PID THROMBIN PAR1 PATHWAY
Negative regulation of phosphoprotein-phosphatase activity
Golgi vesicle transport
Normalized relative abundance
2
1
0
−1
−2
B
D0
R-HSA-5653656: Vesicle-mediated transport
GO:0016071: mRNA metabolic process
R-HSA-8953854: Metabolism of RNA
GO:0030155: regulation of cell adhesion
GO:0006897: endocytosis
GO:0046700: heterocycle catabolic process
GO:0051603: proteolysis involved in cellular protein catabolic process
GO:0009896: positive regulation of catabolic process
R-HSA-8873719: RAB geranylgeranylation
WP4341: Non-genomic actions of 1,25 dihydroxyvitamin D3
GO:0055086: nucleobase-containing small molecule metabolic process
GO:0032753: positive regulation of interleukin-4 production
GO:0001819: positive regulation of cytokine production
GO:0090407: organophosphate biosynthetic process
GO:0006913: nucleocytoplasmic transport
GO:0048193: Golgi vesicle transport
GO:0022407: regulation of cell-cell adhesion
GO:0006470: protein dephosphorylation
WP1541: Energy metabolism
WP3888: VEGFA-VEGFR2 signaling pathway
0 2 4 6 8 10
-log10(P)
C
D28
GO:0055086: nucleobase-containing small molecule metabolic process
GO:0090407: organophosphate biosynthetic process
GO:0016482: cytosolic transport
GO:0010035: response to inorganic substance
GO:0010256: endomembrane system organization
GO:0006457: protein folding
GO:0016050: vesicle organization
GO:0048193: Golgi vesicle transport
GO:0042176: regulation of protein catabolic process
R-HSA-199991: Membrane Trafficking
GO:1903651: positive regulation of cytoplasmic transport
GO:0097435: supramolecular fiber organization
GO:0006979: response to oxidative stress
GO:0042060: wound healing
GO:0018208: peptidyl-proline modification
GO:0008360: regulation of cell shape
GO:0007034: vacuolar transport
GO:0006914: autophagy
GO:0032535: regulation of cellular component size
GO:1902117: positive regulation of organelle assembly
0 1 2 3 4 5 6 7
-log10(P)
D
D57
R-HSA-1428517: The citric acid (TCA) cycle and respiratory electron transport
R-HSA-71406: Pyruvate metabolism and Citric Acid (TCA) cycle
GO:0007005: mitochondrion organization
GO:0009117: nucleotide metabolic process
GO:0016226: iron-sulfur cluster assembly
GO:0000960: regulation of mitochondrial RNA catabolic process
R-HSA-389661: Glyoxylate metabolism and glycine degradation
WP4932: 7q11.23 copy number variation syndrome
GO:1990778: protein localization to cell periphery
WP4519: Cerebral organic acidurias, including diseases
R-HSA-416572: Sema4D induced cell migration and growth-cone collapse
R-HSA-1268020: Mitochondrial protein import
R-HSA-6809371: Formation of the cornified envelope
GO:0032787: monocarboxylic acid metabolic process
GO:0032990: cell part morphogenesis
GO:0032528: microvillus organization
GO:0030162: regulation of proteolysis
GO:0030855: epithelial cell differentiation
R-HSA-1474290: Collagen formation
WP4921: Mitochondrial complex III assembly
0 5 10 15
-log10(P)
E
Estiamted Proportion
1.00 0.75 0.50 0.25 0.00
Time
Group
D0 D28 D57 D0 D28 D57 D0 D28 D57
Group 0 Group 1 Group 2
Cell type
B cells naive
B cells memory
Plasma cells
T cells CD8
T cells CD4 naive
T cells CD4 memory resting
T cells CD4 memory activated
T cells follicular helper
T cells regulatory (Tregs)
T cells gamma delta
NK cells resting
NK cells activated
Monocytes
Macrophages M0
Macrophages M1
Macrophages M2
Dendritic cells resting
Dendritic cells activated
Mast cells resting
Mast cells activated
F
Group Group 0 Group 1 Group 2
Proportion
0.15 0.10 0.05 0.00
* * ** * *
B cells naive
B cells memory
Plasma cells
T cells CD8
T cells CD4 naive
T cells CD4 memory resting
T cells CD4 memory activated
T cells follicular helper
T cells regulatory (Tregs)
T cells gamma delta
NK cells resting
NK cells activated
Monocytes
Macrophages M0
Macrophages M1
Macrophages M2
Dendritic cells resting
Dendritic cells activated
Mast cells resting
Mast cells activated
Cell type
G
Estiamted Proportion
1.00 0.75 0.50 0.25 0.00
D0_Group 0
D0_Group 1
D0_Group 2
D28_Group 0
D28_Group 1
D28_Group 2
D57_Group 0
D57_Group 1
D57_Group 2

Figure S5

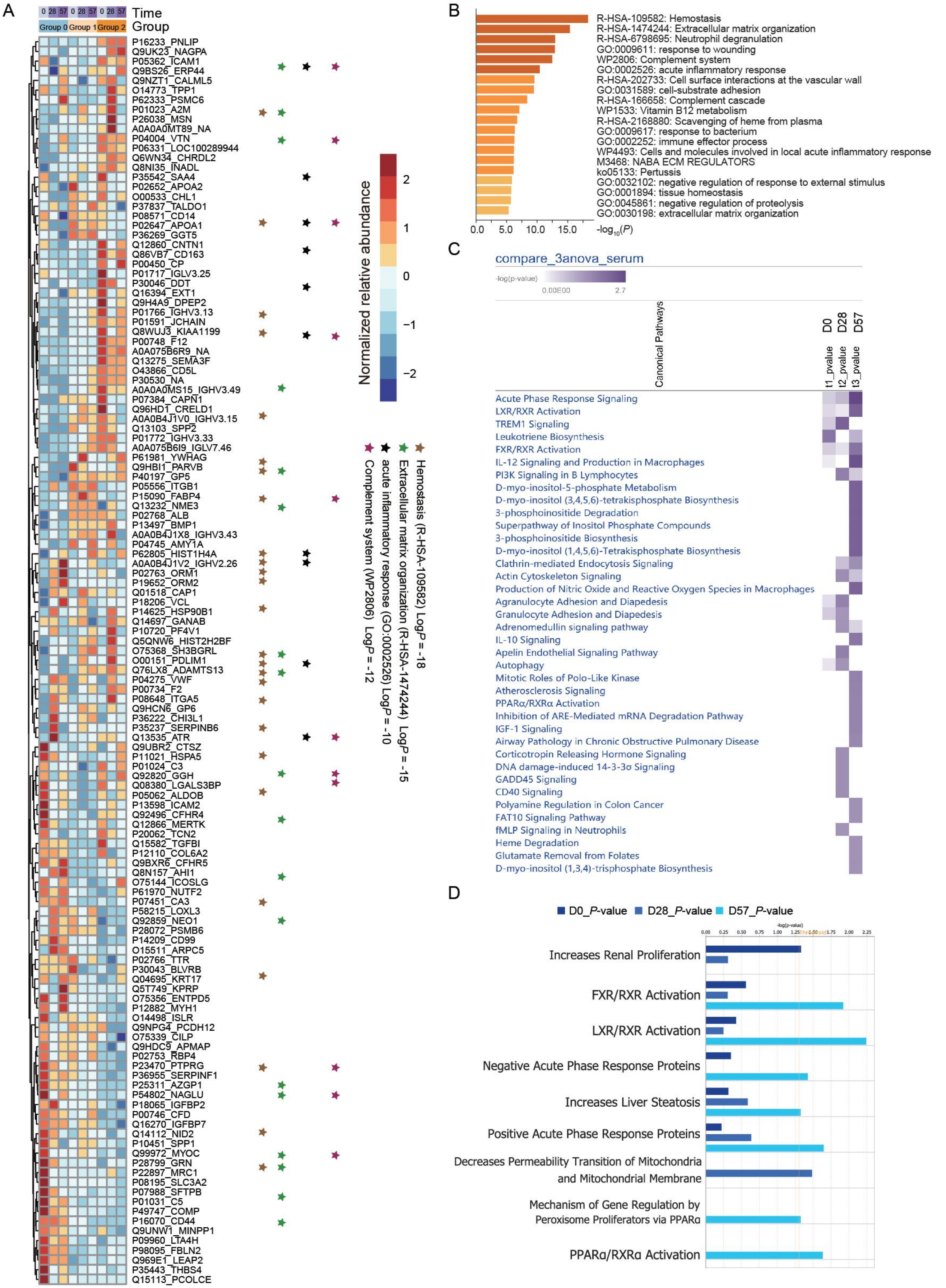
A
Time
Group
Group 0
Group 1
Group 2
Normalized relative abundance
Hemostasis (R-HSA-109582) LogP = -18
Extracellular matrix organization (R-HSA-1474244) LogP = -15
acute inflammatory response (GO:0002526) LogP = -10
Complement system (WP2806) LogP = -12
B
R-HSA-109582: Hemostasis
R-HSA-1474244: Extracellular matrix organization
R-HSA-6798695: Neutrophil degranulation
GO:0009611: response to wounding
WP2806: Complement system
GO:0002526: acute inflammatory response
R-HSA-202733: Cell surface interactions at the vascular wall
GO:0031589: cell-substrate adhesion
R-HSA-166658: Complement cascade
WP1533: Vitamin B12 metabolism
R-HSA-2168880: Scavenging of heme from plasma
GO:0009617: response to bacterium
GO:0002252: immune effector process
WP4493: Cells and molecules involved in local acute inflammatory response
M3468: NABA ECM REGULATORS
ko05133: Pertussis
GO:0032102: negative regulation of response to external stimulus
GO:0001894: tissue homeostasis
GO:0045861: negative regulation of proteolysis
GO:0030198: extracellular matrix organization
-log10(P)
C
compare_3anova_serum
Canonical Pathways
D0
D28
D57
Acute Phase Response Signaling
LXR/RXR Activation
TREM1 Signaling
Leukotriene Biosynthesis
FXR/RXR Activation
IL-12 Signaling and Production in Macrophages
PI3K Signaling in B Lymphocytes
Production of Nitric Oxide and Reactive Oxygen Species in Macrophages
Agranulocyte Adhesion and Diapedesis
Granulocyte Adhesion and Diapedesis
IL-10 Signaling
Autophagy
PPARα/RXRα Activation
IGF-1 Signaling
CD40 Signaling
Heme Degradation
D
D0_P-value
D28_P-value
D57_P-value
Increases Renal Proliferation
FXR/RXR Activation
LXR/RXR Activation
Negative Acute Phase Response Proteins
Increases Liver Steatosis
Positive Acute Phase Response Proteins
Decreases Permeability Transition of Mitochondria and Mitochondrial Membrane
Mechanism of Gene Regulation by Peroxisome Proliferators via PPARα
PPARα/RXRα Activation

Figure S6

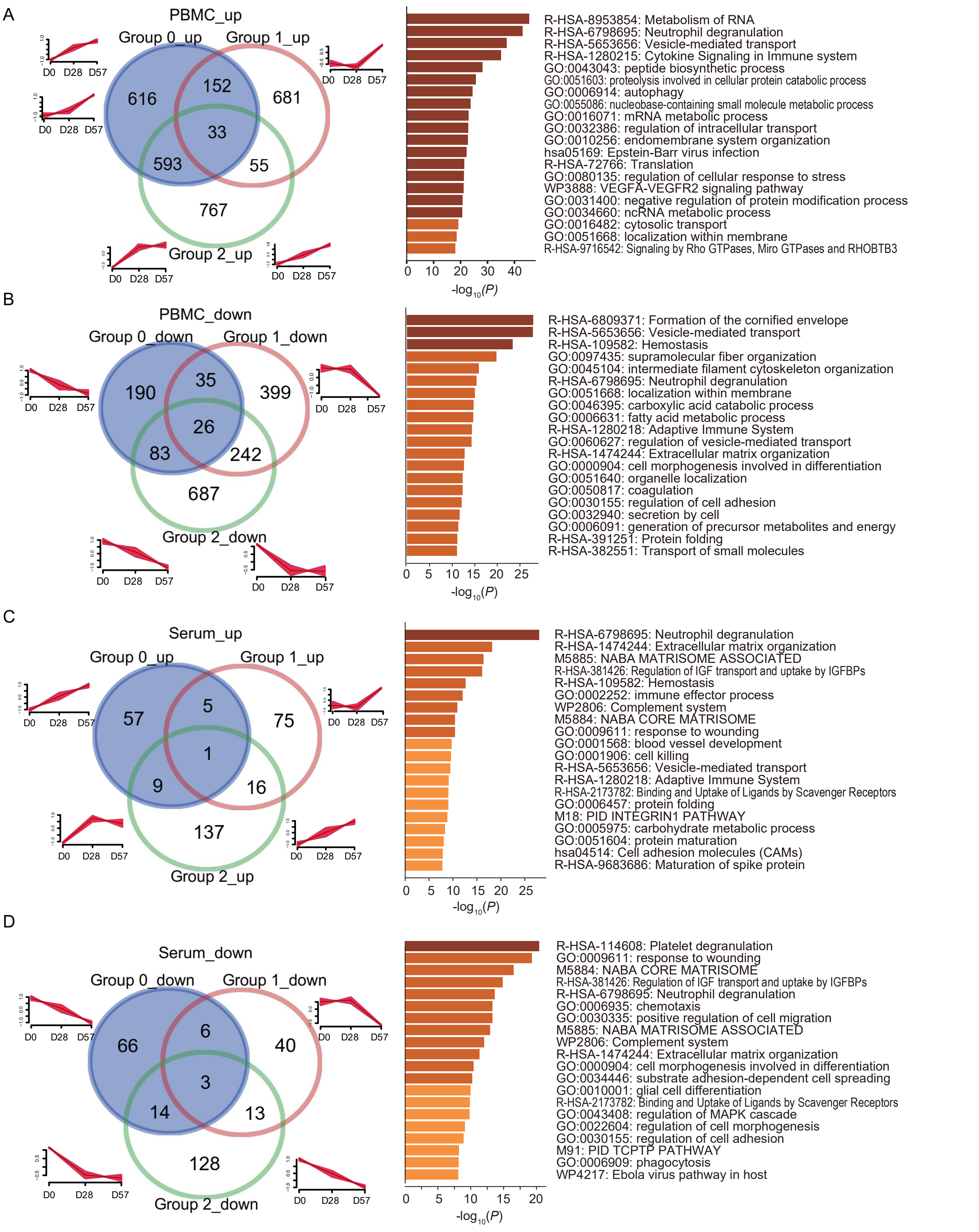
A
PBMC_up
Group 0_up
Group 1_up
Group 2_up
616
152
681
33
593
55
767
R-HSA-8953854: Metabolism of RNA
R-HSA-6798695: Neutrophil degranulation
R-HSA-5653656: Vesicle-mediated transport
R-HSA-1280215: Cytokine Signaling in Immune system
GO:0043043: peptide biosynthetic process
GO:0051603: proteolysis involved in cellular protein catabolic process
GO:0006914: autophagy
GO:0055086: nucleobase-containing small molecule metabolic process
GO:0016071: mRNA metabolic process
GO:0032386: regulation of intracellular transport
GO:0010256: endomembrane system organization
hsa05169: Epstein-Barr virus infection
R-HSA-72766: Translation
GO:0080135: regulation of cellular response to stress
WP3888: VEGFA-VEGFR2 signaling pathway
GO:0031400: negative regulation of protein modification process
GO:0034660: ncRNA metabolic process
GO:0016482: cytosolic transport
GO:0051668: localization within membrane
R-HSA-9716542: Signaling by Rho GTPases, Miro GTPases and RHOBTB3
-log10(P)
B
PBMC_down
Group 0_down
Group 1_down
Group 2_down
190
35
399
26
83
242
687
R-HSA-6809371: Formation of the cornified envelope
R-HSA-5653656: Vesicle-mediated transport
R-HSA-109582: Hemostasis
GO:0097435: supramolecular fiber organization
GO:0045104: intermediate filament cytoskeleton organization
R-HSA-6798695: Neutrophil degranulation
GO:0051668: localization within membrane
GO:0046395: carboxylic acid catabolic process
GO:0006631: fatty acid metabolic process
R-HSA-1280218: Adaptive Immune System
GO:0060627: regulation of vesicle-mediated transport
R-HSA-1474244: Extracellular matrix organization
GO:0000904: cell morphogenesis involved in differentiation
GO:0051640: organelle localization
GO:0050817: coagulation
GO:0030155: regulation of cell adhesion
GO:0032940: secretion by cell
GO:0006091: generation of precursor metabolites and energy
R-HSA-391251: Protein folding
R-HSA-382551: Transport of small molecules
-log10(P)
C
Serum_up
Group 0_up
Group 1_up
Group 2_up
57
5
75
1
9
16
137
R-HSA-6798695: Neutrophil degranulation
R-HSA-1474244: Extracellular matrix organization
M5885: NABA MATRISOME ASSOCIATED
R-HSA-381426: Regulation of IGF transport and uptake by IGFBPs
R-HSA-109582: Hemostasis
GO:0002252: immune effector process
WP2806: Complement system
M5884: NABA CORE MATRISOME
GO:0009611: response to wounding
GO:0001568: blood vessel development
GO:0001906: cell killing
R-HSA-5653656: Vesicle-mediated transport
R-HSA-1280218: Adaptive Immune System
R-HSA-2173782: Binding and Uptake of Ligands by Scavenger Receptors
GO:0006457: protein folding
M18: PID INTEGRIN1 PATHWAY
GO:0005975: carbohydrate metabolic process
GO:0051604: protein maturation
hsa04514: Cell adhesion molecules (CAMs)
R-HSA-9683686: Maturation of spike protein
-log10(P)
D
Serum_down
Group 0_down
Group 1_down
Group 2_down
66
6
40
3
14
13
128
R-HSA-114608: Platelet degranulation
GO:0009611: response to wounding
M5884: NABA CORE MATRISOME
R-HSA-381426: Regulation of IGF transport and uptake by IGFBPs
R-HSA-6798695: Neutrophil degranulation
GO:0006935: chemotaxis
GO:0030335: positive regulation of cell migration
M5885: NABA MATRISOME ASSOCIATED
WP2806: Complement system
R-HSA-1474244: Extracellular matrix organization
GO:0000904: cell morphogenesis involved in differentiation
GO:0034446: substrate adhesion-dependent cell spreading
GO:0010001: glial cell differentiation
R-HSA-2173782: Binding and Uptake of Ligands by Scavenger Receptors
GO:0043408: regulation of MAPK cascade
GO:0022604: regulation of cell morphogenesis
GO:0030155: regulation of cell adhesion
M91: PID TCPTP PATHWAY
GO:0006909: phagocytosis
WP4217: Ebola virus pathway in host
-log10(P)

Figure S7

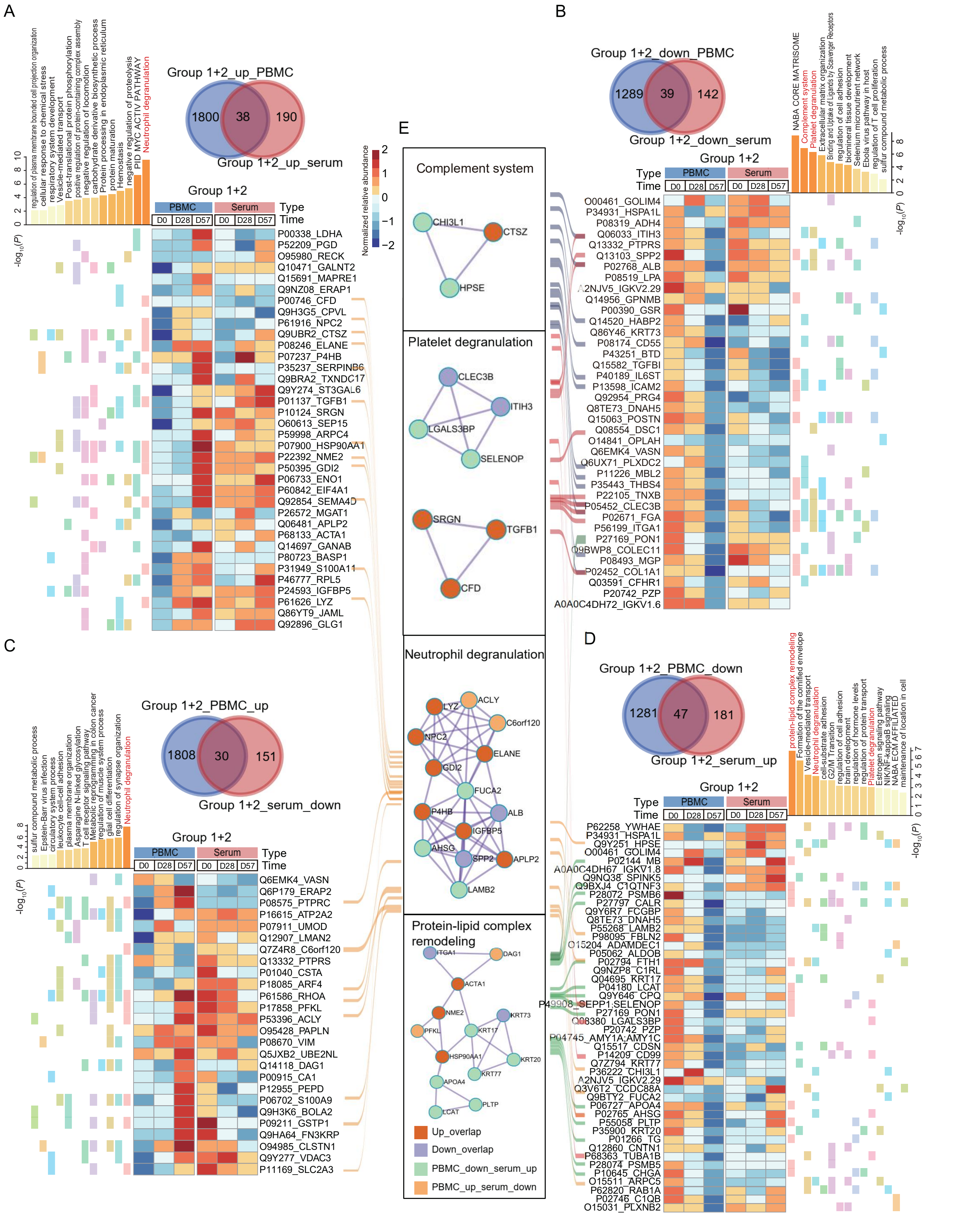

A
Group 1+2_up_PBMC
1800
38
190
Group 1+2_up_serum
Group 1+2
PBMC
Serum
Type
Time
D0
D28
D57
B
Group 1+2_down_PBMC
1289
39
142
Group 1+2_down_serum
C
Group 1+2_PBMC_up
1808
30
151
Group 1+2_serum_down
D
Group 1+2_PBMC_down
1281
47
181
Group 1+2_serum_up
E
Complement system
Platelet degranulation
Neutrophil degranulation
Protein-lipid complex remodeling
Normalized relative abundance
Up_overlap
Down_overlap
PBMC_down_serum_up
PBMC_up_serum_down
-log10(P)
